# Therapeutic mechanisms of polysaccharides in the management of rheumatoid arthritis: a comprehensive review

**DOI:** 10.3389/fimmu.2025.1608909

**Published:** 2025-07-01

**Authors:** Wenlong Liu, Youqian Kong, Xiaoyu Wang, Yuanyuan Yang, Qi Yan, Zeguang Li

**Affiliations:** ^1^ Graduate School, Heilongjiang University of Chinese Medicine, Harbin, China; ^2^ First Affiliated Hospital, Heilongjiang University of Chinese Medicine, Harbin, China

**Keywords:** rheumatoid arthritis (RA), polysaccharides, mechanism, immune cells, cytokines

## Abstract

Rheumatoid Arthritis (RA) is a chronic autoimmune disease characterized by synovial inflammation and destruction of articular cartilage and bone, which seriously affects patients’ quality of life. In recent years, with the in-depth research on natural medicines, the application of polysaccharides in the treatment of RA has gradually gained attention due to their unique bioactive components and diverse pharmacological effects. Polysaccharides were reported to exert anti-inflammatory, antioxidant, immunomodulatory, and protective effects on cartilage and bone tissues. This review briefly introduces RA, its etiology and pathogenesis, and the different sources and structures of polysaccharides. It focuses on the mechanisms of polysaccharides in the alleviation of RA, mainly through the modulation of immune cell function, inhibition of inflammation, regulation of gut microbiota, promotion of bone formation and repair, and influence on related pathways. The aim of this review is to summarize the polysaccharides and their mechanisms of action in the alleviation of RA, with a view to providing new ideas for the clinical treatment of RA.

## 1 Introduction

Rheumatoid arthritis (RA) is one of the most common chronic autoimmune diseases (1). Its pathogenesis involves a complex interaction between abnormal activation of the immune system, inflammatory response and gut microbial homeostasis (2). Epidemiology shows that the global prevalence of RA is about 0.5%-1%, and the incidence in women is 2–3 times higher than that in men, which seriously affects the quality of life of patients (3). Once diagnosed, RA requires lifelong treatment (4). It also brings a heavy economic burden to the society. Currently, the treatment strategies for RA mainly include drug therapy, physiotherapy, surgery (5–8). However, in view of its complex pathogenesis and diverse clinical manifestations, the search for safer and more effective treatments has always been a research hotspot in the field of rheumatology (9–11). In recent years, polysaccharides, as a class of natural products with a wide range of biological activities, have gradually attracted attention for their potential in alleviating rheumatoid arthritis (12, 13). The aim of this article is to review the role of polysaccharides and their potential mechanisms in the alleviation of RA, with a view to providing new ideas and strategies for the treatment of RA.

Polysaccharides are a class of macromolecular compounds consisting of multiple monosaccharide molecules linked by glycosidic bonds, which are widely found in plants, animals and microorganisms (14–16). Depending on the source, polysaccharides can be classified as plant polysaccharides, animal polysaccharides and microbial polysaccharides (17–19). Polysaccharides have a variety of biological activities, such as immunomodulation, anti-inflammatory, antioxidant, anti-tumour (20, 21). These activities are closely related to their complex chemical structures. In recent years, more and more studies have shown that polysaccharides can regulate the immune system, inhibit the inflammatory response, promote tissue repair and other pathways to produce therapeutic effects on a variety of diseases, including rheumatoid arthritis (22, 23). The pathogenesis of RA is complex and involves a variety of aspects, such as genetics, the environment, immunity (24, 25). In the pathogenesis of RA, the immune system is abnormally activated and attacks its own synovium, leading to synovial inflammation and joint damage (26, 27). Synovial inflammation further triggers the formation of pannus, which invade the articular cartilage and bone, releasing a variety of inflammatory mediators and proteases and accelerating joint destruction (28, 29). In addition, RA patients suffer from pathological changes such as enhanced oxidative stress and imbalanced cytokine networks, which together contribute to disease progression (30, 31). Therefore, therapeutic strategies for RA need to take multiple aspects into account, including inhibiting the inflammatory response, regulating immune balance, and promoting tissue repair (32, 33).

**Figure 1:**
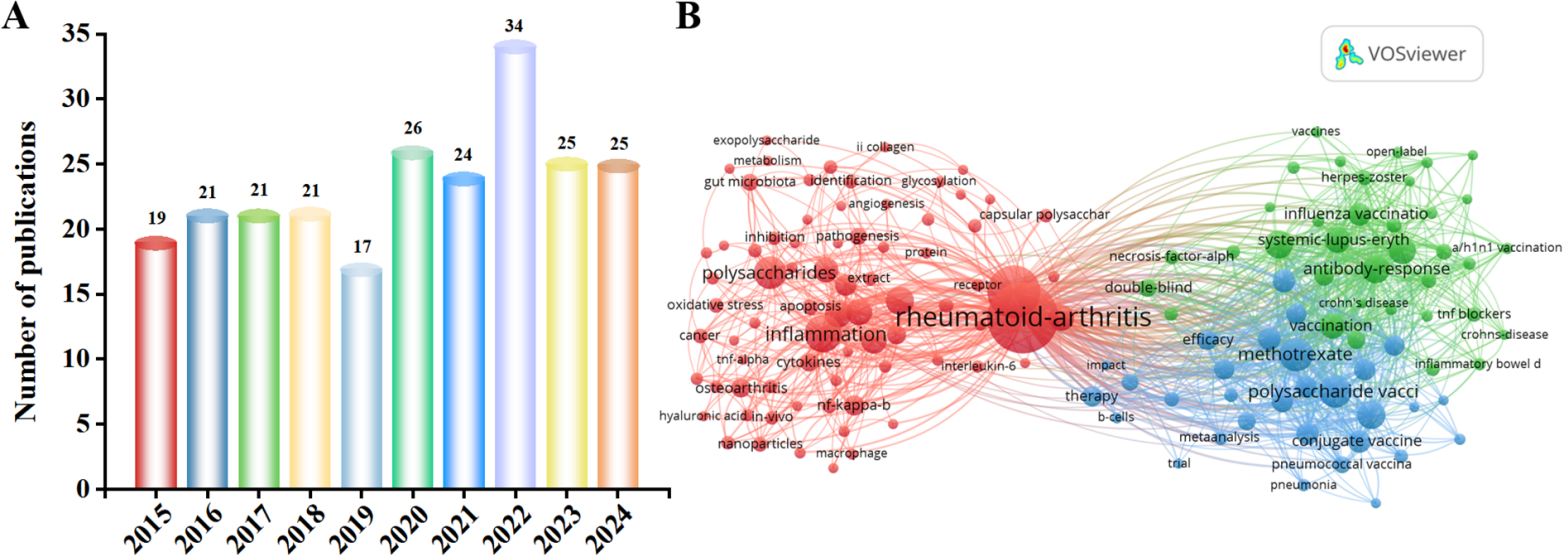
**(A)** Number of polysaccharide- and RA-related papers obtained from the Web of Science core database from 2015 to 2024. **(B)** Keyword co-occurrence map of bibliographic data created using VOSviewer from 2015 to 2024. Specifically, this area remains underexplored from 2015 to 2024. In 2022, polysaccharides for RA were published to a near-decade high of 34 articles, and have remained at 25 articles in 2023 and 2024. Thus, research findings on polysaccharides for RA have been of interest.

Over the past decade, mentions of polysaccharides and RA increased from 19 in 2015 to 25 in 2024 (**Figure 1A**). **Figure 1B** shows the co-occurrence of keywords related to polysaccharides and RA. Among them, there are many keywords related to RA, such as inflammation, Interleukin-6 (IL-6) and B cells. This review provides a reference for further research and application of developing new therapeutic strategies for RA. The aim of this review is to provide a comprehensive overview of current research involving plant polysaccharides, animal polysaccharides and microbial polysaccharides, the therapeutic rheumatological activity of polysaccharides from different sources and their mechanisms, with a particular focus on the relevant signaling pathways for the treatment of RA, NF-κB, PI3K/AKT, JAK/STAT and MAPK. This work also centers around the need for further research to better understand the limitations of clinical therapy with polysaccharides. It also critically examines the challenges associated with its clinical use, especially the safety concerns.

## 2 Methods

This narrative review searched PubMed, Web of Science, SpringerLink and Science Direct databases using keywords and related terms. It used certain keywords, i.e. polysaccharides and RA, and combined these terms with the following keywords: plant, microorganism, animal, immune, inflammatory, oxidative, bone tissue, pathway, etc. The last search was conducted in March 2025. The language of literature search was English and references were selected based on their relevance. Duplicate studies and irrelevant references were excluded, and abstracts of the remaining articles were reviewed to ensure they met the inclusion criteria for the review.

## 3 RA background

### 3.1 Etiology and pathogenesis of rheumatoid arthritis

**Figure 2:**
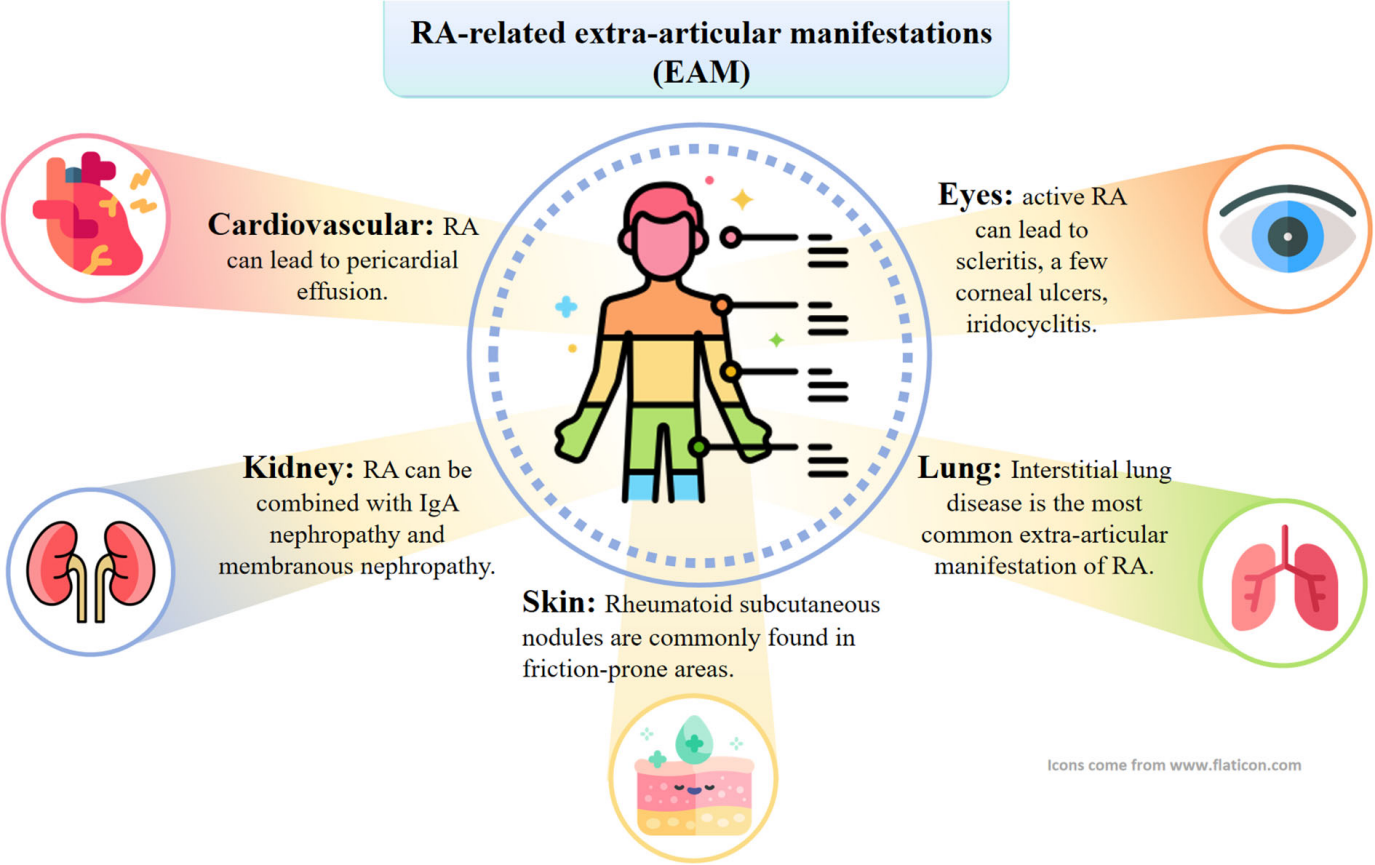
RA can also involve internal organs other than joints. Specifically, this can manifest as interstitial lung lesions, rheumatoid nodules, skin ulcers, and neurologic, cardiac, and eye lesions.

RA is an autoimmune disease that is characterized by focusing on articular cartilage erosion and bone destruction, ultimately leading to joint deformity and loss of function (34). The main clinical manifestations of patients are symmetrical morning stiffness, pain and swelling of multiple joints to varying degrees, accompanied by limited movement, and joint deformity may occur in patients with a longer course of the disease (35, 36). RA-related extra-articular manifestations (EAM) can involve all organ systems, with a wide range of symptoms (37, 38). As shown in **Figure 2**, in addition to intra-articular manifestations, there may be secondary tissue and organ damage such as cardiovascular, pulmonary, renal, ocular, cutaneous ulcer, digestive and neurological disorders (39). Approximately 17.6 million people worldwide suffer from RA, and more than 3 million are disabled. As the population continues to age, the number of RA cases is projected to reach 31.7 million by 2050, increasing burden on society (39). Epidemiological studies have shown that the prevalence of RA in China is 0.42 per cent, affecting about 5 million people, with a male-to-female ratio of about 1:4 (40).

### 3.2 The development and pathogenesis of RA

**Figure 3:**
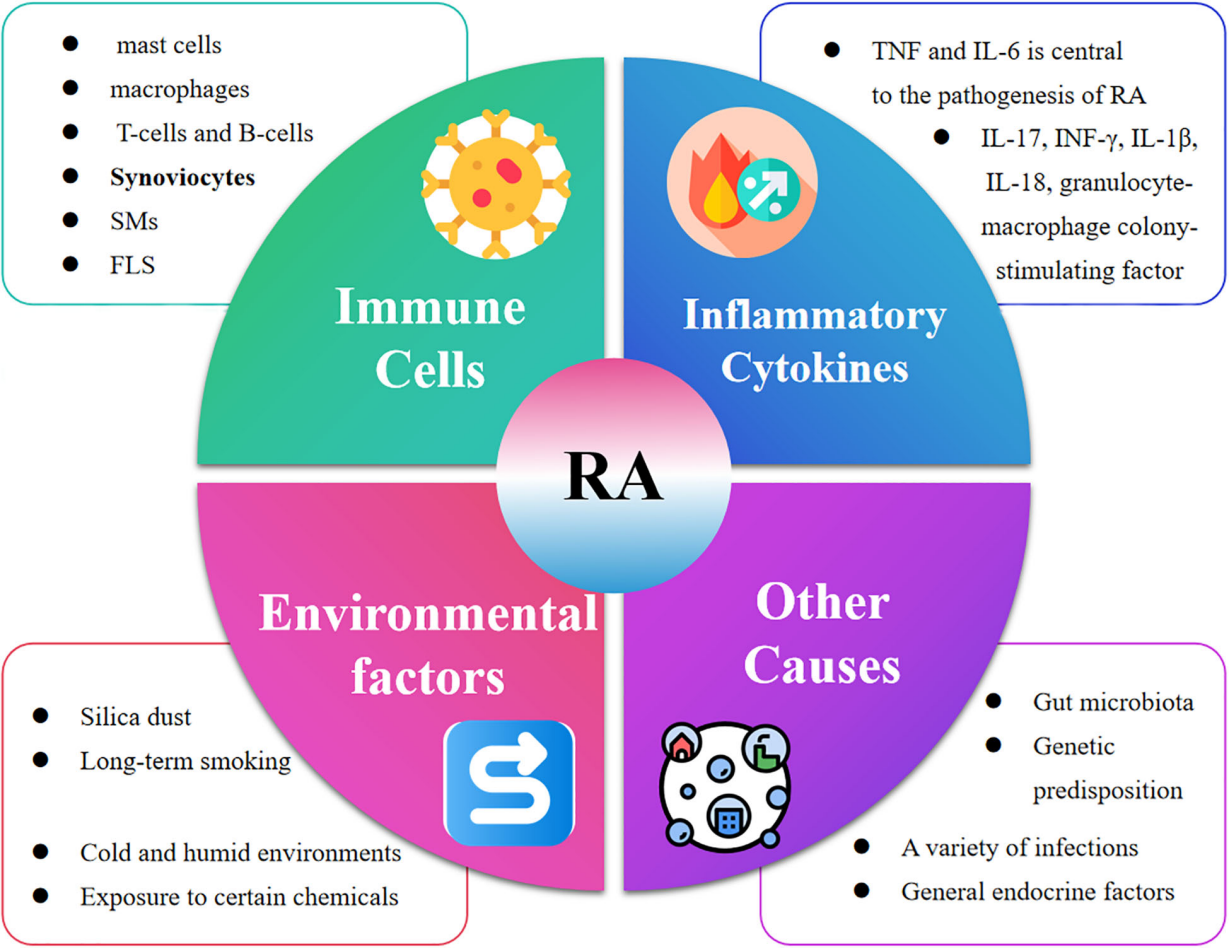
The development of RA is the result of a combination of factors. Specifically, Immune Cells (mast cells, macrophages, T-cells and B-cells, Synoviocytes, SMs, FLS), Inflammatory Cytokines (TNF and IL-6), Environmental factors (Silica dust, Long-term smoking, Cold and humid environments Exposure to certain chemicals and Other Causes (Gut microbiota, Genetic predisposition, A variety of infections, General endocrine factors) jointly accelerated the development of RA.

As shown in **Figure 3**, the pathogenesis of RA is complex and is the result of a combination of factors. An imbalance in the immune system is key. The immune imbalance in rheumatoid arthritis is characterized by T-cell abnormalities and an imbalance of pro-inflammatory/anti-inflammatory factors that trigger joint destruction (41, 42). Articular cartilage and bone are destroyed by inflammatory mechanisms involving a variety of pro-inflammatory factor-secreting cells, including immune cells (e.g., mast cells, macrophages, dendritic cells, T-cells, and B-cells) and synoviocytes (synovial macrophages (SMs) and fibroblast-like synoviocytes (FLS)) (43). Similarly, inflammatory cytokines play a key role in the development of RA, the synovial inflammatory response and bone destruction (44, 45). Among them, the involvement of TNF and IL-6 is central to the pathogenesis of RA, and other cytokines such as IL-17, IFN-γ, IL-1β, IL-18, and granulocyte-macrophage colony-stimulating factor (GM-CSF) also play important roles (46). These cytokines, together with immune cells and autoantibodies, induce and maintain joint inflammation in RA, ultimately leading to cartilage and bone damage in the affected joints (**Table 1**). RA has a recognized genetic component, and studies have shown that in identical twins, if one twin has RA, the other has a probability of developing the disease of about 15 per cent. Certain genes are associated with the development of RA, such as HLA-DR4, which affects the immune system’s recognition and response, increasing the risk of the disease (47). In addition, a variety of infections are associated with the development of RA, such as EBV, *Chlamydia pneumoniae*, and *Helicobacter pylori*. Infections may activate the immune system, and some components of pathogens are similar to the body’s own antigens, so that the immune system attacks the pathogen by attacking its own tissues by mistake, i.e., a molecular mimicry mechanism (48). General endocrine factors can also trigger rheumatoid arthritis, such as sex hormones. The incidence of RA is higher in women than in men. The risk of RA in women particularly increases during pregnancy, postpartum and around the time of menopause due to hormonal fluctuations (49). Estrogen regulates immune cell function, affects cytokine secretion, alters the immune balance and increases the susceptibility of RA. Lifestyle habits and environment can trigger rheumatoid arthritis (49). For example, long-term smoking disrupts the immune balance in the lungs and produces inflammatory factors (TNF-α, IL-8, CCL20), increasing the likelihood of RA onset (50). Cold and humid environments can affect blood circulation and immune function, increasing the risk of developing RA (51). Exposure of the body to certain chemicals, such as silica dust, may also trigger RA (52). With advances in high-throughput sequencing, it has been widely recognized that the host microbiota, particularly the gut microbiota, plays a key role in the pathogenesis and progression of RA (53). The gut microbiota and gut-associated lymphoid tissues work together to maintain immune homeostasis within the host and can serve as an indicator of host health status, and if their interactions are disrupted, they can have an impact on mucosal and systemic immunity and lead to the development of a variety of inflammatory and autoimmune diseases (54). Therefore, further elucidation of the pathogenesis of RA and promotion of RA-related drug research are of great significance in improving treatment efficacy of RA.

**Table 1:** Multiple immune cells and cytokines play important roles in RA intrinsic and adaptive immunity and its development.

Type		Major role	References
Immune cells	T cells	The CD4+ T cell subset of T cells is the dominant cell type in the pathogenesis of RA. Regulatory T cells and Th17-dominant Th can stimulate the differentiation of a variety of cytokines, and the imbalance of Th1/Th2 and Th17/regulatory T cells is key to the development of RA.	(109)
	B cells	It can activate macrophages and osteoclasts, act as antigen-presenting cells to activate T cells, and secrete autoantibodies and inflammatory factors such as TNF-α, IFN-γ, IL-6, IL-1β, IL-17, IL-18, etc.	(110)
	Macrophages	Activatable macrophages drive synovial inflammation by secreting cytokines, and their degree of infiltration in the synovium correlates with the degree of joint erosion.	(111)
	Dendritic cells	Mature dendritic cells effectively activate the initial T cells, which are located at the center of initiating, regulating, and maintaining the immune response.	(112)
	Neutrophils	Release of multiple degradative enzymes, inflammatory factors and reactive oxygen species leads to vascular endothelial dysfunction and tissue damage, and secretion of inflammatory factors such as IL-1, IL-6, IL-12, TGF-β and TNF.	(113)
	Mast cells	It increases vascular permeability, activates synoviocytes, promotes vascular proliferation, and participates in matrix remodeling, which in turn leads to the destruction of cartilage and bone.	(114)
Synovial cells	SMs	A variety of cytokines can be secreted to regulate and mediate immune response processes. They promote inflammatory responses, enhance activation and migration of immune cells, and promote wound healing.	(115)
	FLS	Responsible for producing a variety of cytokines that may significantly reduce inflammation and damage in the joints.	(116)
Cytokines	TNF	TNF inhibitors prevent TNF from binding to its receptor and inhibit the inflammatory cascade, reducing synovial inflammation and joint damage.	(117)
	IL-6	IL-6 plays a major role in rheumatoid arthritis by mediating the inflammatory response, exacerbating joint damage and promoting the autoimmune response.	(118)
	IL-17	IL-17 synergizes and releases a variety of pro-inflammatory cytokines (e.g., IL-1β, IL-6, and TNF-α), stimulates a variety of chemokine ligands involved in the formation of vascular opacities, exacerbates synovial inflammation, and destroys articular bone and cartilage.	(119)
	IFN-γ	Regulate the function of T-cells, B-cells and other immune cells, correct immune imbalance, inhibit autoantibody production and abnormal attack of immune cells on joint tissues.	(120)
	IL-1β	IL-1β can be synthesized and secreted by a variety of cells, such as monocyte-macrophages, fibroblasts, B-lymphocytes, natural killer cells, and smooth muscle cells, among which monocyte-macrophages are predominant.	(121)
	IL-18	It plays an important role in the pathogenesis of rheumatoid arthritis by inducing IFN-γ production through multiple pathways.	(122)
	GM-CSF	GM-CSF promotes the activation, differentiation, survival, and proliferation of monocytes and macrophages transported in synovial joints of inflammatory tissue RA, as well as resident tissue macrophages.	(123)

## 4 Polysaccharide and RA

### 4.1 Sources of polysaccharides

Polysaccharides are macromolecular compounds consisting of multiple monosaccharide molecules linked by glycosidic bonds and are widely found in living organisms. Depending on the source, polysaccharides can be classified into three main categories: plant polysaccharides, animal polysaccharides and microbial polysaccharides. As shown in **Table 2**, the preparation, structure, activity, and references of polysaccharides from different origins were listed. In addition, polysaccharides from different sources (plants, animals, microorganisms) exhibit unique mechanisms in RA therapy due to differences in chemical structure and targets of action. Plant polysaccharides are mainly heteropolysaccharides, which often contain hydroxyl and carboxyl groups, have high molecular weights, and some of them have helical conformations (55). Plant polysaccharides can inhibit the autoimmune response of RA by regulating Th17/Treg (56). Animal polysaccharides act directly on the joint microenvironment by virtue of sulfation modification to alleviate acute inflammation and cartilage damage (57). Microbial polysaccharides regulate systemic immune homeostasis through the “gut-joint axis” and are suitable for RA associated with gut dysbiosis (58).

**Table 2:** Polysaccharides of different origins.

Origins		Preparation	Structure	Activity	References
Plant	*Cynanchum Auriculatum*	α-Amylase→glucoamylase→anhydrous ethanol→DEAE Sepharose Fast Flow column→dialysis	The backbone included β-1,4-Manp, β-1,4,6-Manp, β-1,4-Glcp and β-1,4,6-Glcp residues, with branches at the O-6 position of β-1,4,6-Manp and β-1,4,6-Glcp residues, consisting of α-T-Araf, α-1,5-Araf, α-1,2,5-Araf, α-1,3,5-Araf, T-Xylp,1,4-Xylp, β-T-Manp and β-T-Galp residues.	Immunomodulatory activity	(124)
	*Typha angustifolia*	DEAE-52 cellulose chromatography	PTA-1 comprises glucose (100%) with α-(1→3) glycosidic bonds, and PTA-2 comprises glucose (66.7%) and rhamnose (33.3%) formed by β-(1→3) glycosidic bonds.	Anti-Inflammatory Activity	(125)
	Pumpkin	ethanol/ammonium sulfate system→DEAE cellulose-52 anion exchange chromatography column	The backbone of ATPS-PP-1 comprised of (1→3)-linked-Glcp having branching points at O-3 of (1→3,4)-linked-Glcp with terminal Glcp as side chain. ATPS-PP-2, on the other hand, comprised of 1→Glcp, (1→3)-linked-Galp, (1→6)-linked-Glcp, (1→3,6)-linked-Glcp and (1→4)-linked-Glcp as backbone.	Hypoglycemic Activity	(126)
	*Millettia Speciosa* Champ	decolorization and deproteinization by AB-8 and Sevag method→DEAE-52 cellulose column→Sephadex G-100 column	The backbones of MSCP1 were composed of 1,4-linked-α-D-Glcp, 1,4-linked-α-D-Xylp and 1,4,6-linked-β-D-Glcp. The branch chain T-linked-α-D-Glcp was confirmed to be attached at C-6 of 1,4,6-linked-β-D-Glcp.	immunomodulatory activity	(127)
	*Phragmites rhizoma*	Alcohol Precipitation→Sevag method→DEAE-52 cellulose chromatography column→Sephadex G-100 column	Three sugar residues, →3)-β-D-GalpA-(1→, →2, 3)-α-L-Fucp-(1→and α-L-Fucp (4SO3−)-(1→PRP-2	Anti-inflammatory activity	(128)
	*Bangia fuscopurpurea*	Alcohol Precipitation→neutral protease→DEAE-Sepharose Fast Flow→High-performance gel permeation chromatography (HPGPC)→Gas chromatography-mass spectrometry	Repeating 5-α-l-Araf-1→(4-α-d-Glcp-1)4→4,6-β-d-Manp-1 units, and the side chains consisted of repeating β-d-Galp-1→(4-β-d-Galp-1)4→4,6-β- d-Galp-1→3,4-α-l-Rhap, β-l-Arap-1→(3-β-d-Galp-1)3, and β-l-Arap-1 units	Antitumor activity	(129)
Animal	*Hemicentrotus pulcherrimus*	distilled water→papain and Sevag reagent→DEAE-52→Sepharose CL-2B	featuring a linear backbone of 1,4-linked α-d-glucose with 1,6-α-d-glucose and 1,6-α-D-glucuronic acid side chains grafted on the backbone in an alternating pattern	Antitumor activity	(130)
	*Holothuria nobilis*	anion-exchange FPA98 resin column→HPGPC→Sephadex G25 column→Superdex peptide 10/300 GL column→Dionex IonPac™ AS11-HC column	{4)-[d-GalNAcR1-(α1,2)-l-Fuc3S-(α1,3)-]-d-GlcUA-(β1,3)-d-GalNAc4S6S-(β1},m-{4)-[l-FucR2-(α1,3)-]-d-GlcUA-(β1,3)-d-GalNAc4S6S-(β1},n	Anticoagulant and anti-iXase activities	(131)
	*Lysastrosoma anthosticta*	Gas liquid chromatography of alditol acetates→Gel chromatography→Sephadex G-15 column	[→3)-β-D-GalNAc4S-(1→4)-α-L-IdoA2S3S-(1→]n	Anticoagulant activity	(132)
	Mussel	Enzymatic→anhydrous ethanol→DEAE Sepharose Fast Flow→Polysaccharide gel purification system→HPGPC	→4)-α-D-Glcp-(1→, and the end group α-D-Glcp-(1→and α-D-Glcp-(1→6) -α-D-Glcp-(1→pass→4,6)-α-D-Glcp-(1→	Antioxidant activity	(133)
	*Coelomactra antiquata*	Enzymatic hydrolysis→Ion exchange→Ethanol precipitation→HPGPC	→4)-α-IdoA2S-(1→4)-α-GlcNS3S6S(or GlcNS6S)-(1→4)-β-GlcA-(1→4)-α-GlcNS6S (or GlcNAC)-(1→	Anticoagulant and Fibrinolytic Activities	(134)
Microorganism	*Ganoderma lucidum* spore	Alcohol Precipitation→α-Amylase→Chromatography→Desalination	Glc-(1→3)-Glc-(1→3)-Glc-(1→6)-Glc, Glc-(1→6)-Glc-(1→6)-Glc-(1→6)-Glc and Glc-(1→3)-Glc-(1→3)-Glc-(1→3)-Glc-(1→3)-Glc	immunomodulatory activity	(135)
	*Flammulina velutipes*	Alcohol Precipitation→anhydrous ethanol, acetone, and petroleum ether→Sevag reagent and dialysis bags→DEAE-52 cellulose column→Sephadex G-100 column	CHFVP-1: the backbone of→6)-α-D-Galp-(1→and the branch of Galp by an →3,6)-α-D-Manp-(1→attached with T-β-D-Glcp or t-α-L-Fucp side chains. CHFVP-2: the construction of →6)-β-D-Glcp-(1→and T-β-D-Glcp.	Coagulant activity	(136)
	*Cordyceps militaris* (L.) Link	NaOH→HCl→cellulose membrane→Q-Sepharose™ Fast Flow column→ Sephacryl S400HR column	CM3I: composed of→4)α-D-Glcp(1→glycosyls and differed from starch due to the presence of→4,6)β-D-Glcp(1→glycosyls. CM3II: composed of →4)-β-D-Manp(1→6)-α-D-Manp(1→6)-β-D-Manp(1→linked glycosyls, and especially the presence of O-methyl.	Anti-atherosclerotic activity	(137)
	*Ramaria flaccida* (Fr.) Quél	Alcohol Precipitation→DEAE-52 cellulose column→GC-MS	The main chain of RF−1 consisted of (1→6, 2)−α−D−galactopyranose and (1→6, 4)−​α−D−glucopyranose. One of the branched chains was linked to 4−O of the main glucose chain by (1→6)−α−D−glucopyranose and next linked by one (→4)−β−D−glucopyranose. The other two branched chains were both linked to 2−O of the main glucose chain by one (→4)−β−D−glucopyranose.	Antitumor activity	(138)
	*Boletus reticulatus* Schaeff	Alcohol Precipitation→DEAE cellulose→Dialysis→HPGPC→HPLC→GC-MS	1,6-linked α-D-galactose and 1,2,6-linked α-D-galactose which branches were mainly composed of a terminal 4-linked β-D-glucose	Antitumor activity	(139)

### 4.2 Mechanisms associated with the alleviation of RA by polysaccharides

**Figure 4:**
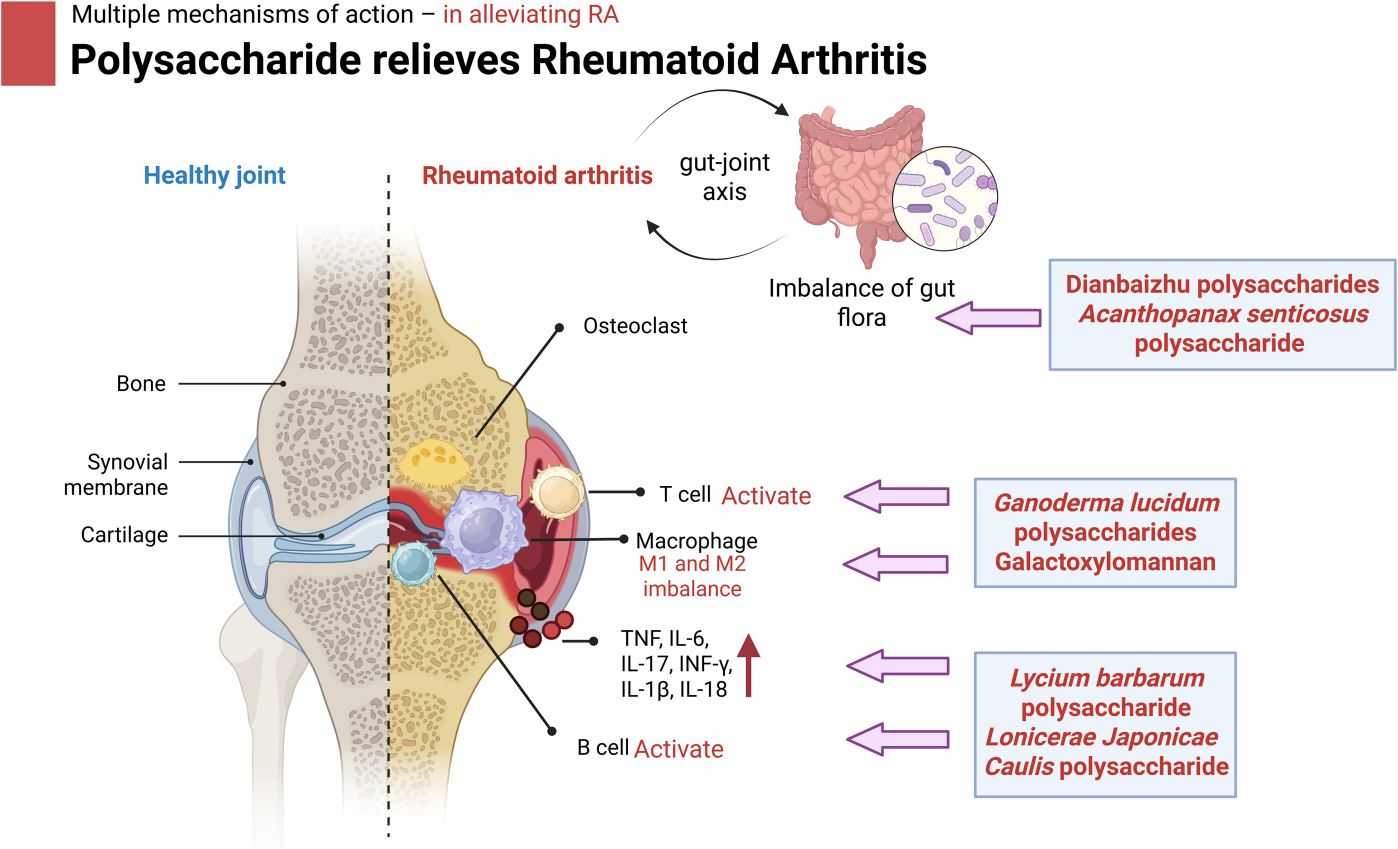
Mechanism of action of polysaccharides to alleviate OA in mouse. Specifically, polysaccharides of different origins treat RA progression by modulating immune macrophages, inhibiting inflammatory factors, improving the gut microbiota and its metabolites, promoting cartilage and bone tissue repair, and modulating related pathways. Image created with BioRender.com, with permission.

Polysaccharides, as natural active ingredients, have a variety of mechanisms of action to alleviate RA, which may provide new ideas for the treatment of RA, such as by regulating immune cell function, inhibiting inflammation, regulating the gut microbiota, promoting bone formation and repair and related pathways (**Table 3**). As shown in **Figure 4**, polysaccharides exhibit multiple mechanisms of action in alleviating RA.

**Table 3:** Experimental models of rheumatoid arthritis.

Polysaccharides		Model	Dose	Mechanisms	Reference
*Ephedra sinica*	pure polysaccharide ESP-B4	a Freund's complete adjuvant (FCA)-induced RA model in rats; THP-1 cells, RAW264.7 cells and PBMC cells	200 and 100 mg/kg/day	ESP-B4 significantly improves all parameters of inflammation. Treats RA by inhibiting the TLR4 signaling pathway, which reduces the release of inflammatory factors and cytokines.	(140)
*Angelica sinensis*	Angelica sinensis polysaccharide ASP-2pb	type-II collagen induced arthritis (CIA) modeling	400 mg/kg	ASP-2pb affects specific gut bacteria as well as metabolites produced by gut bacteria in the process of alleviating RA.	(141)
*Anoectochilus roxburghii*	ARP	CIA mouse model	120 mg / kg	ARP significantly inhibited the activation of the NF-κB pathway by inhibiting the phosphorylation of IκB and p65, thereby down-regulating the mRNA expression of IL-1β and IL-6 in LPS.	(142)
*Holothuria leucospilota*	*Holothuria leucospilota* polysaccharides (HLP)	intraperitoneally immunosuppressive model	80 mg/kg	HLP improves the immune factors, T-cell markers, and Toll-like receptors (TLR) pathway-related proteins expression. HLP increases the short-chain fatty acids concentration and regulates the gut microbiota composition.	(143)
*Streptococcus pararoseus*	*Streptococcus pararoseus* polysaccharide (SPP)	CIA mouse model	400 mg/kg	The SPP intervention increased the relative abundance of beneficial bacteria that have the potential to degrade fungal polysaccharides or produce short-chain fatty acids.	(144)
Wu-tou decoction	Wu-tou decoction polysaccharides (PS)	a model of adjuvant-induced arthritis (AIA) in rats	9.8 g crude drug/kg/day	PS may be able to control the disruption of the intestinal microbiota and improve the intestinal environment in model animals, thereby influencing the absorption and metabolism of SM	(145)
	Gelatin	Raw264.7 cells and chondrocyte cells	Gelatin as a carrier	Gel/FA-PDA@Leon hydrogel inhibits inflammatory responses and protects chondrocytes from ferritin phagocytosis/iron death by down-regulating the JAK2/STAT3 signaling pathway in macrophages.	(146)
	chitosan	a FCA-induced RA model in rats	20 mg/kg	Chitosan nanoparticles (Q-NPs) containing quercetin significantly reduced ankle diameter, oxidative stress markers, and inflammatory cytokines such as TNF-α and IL-6.	(147)
	chondroitin sulfate	Caudal intradermal injection of heat inactivated Mycobacterium tuberculosis incomplete Freund's adjuvant suspension	300 mg/kg	Chondroitin sulfate with glucosamine significantly reduced paw swelling and inflammatory markers while altering intestinal flora by inhibiting LPS-producing bacteria and the TLR-4/NF-κB pathway.	(148)

#### 4.2.1 Regulation of immune cell function

RA is an autoimmune disease mainly characterized by chronic synovial inflammation, and its pathogenesis is complex, involving the abnormal activation of multiple immune cells and cytokines. In recent years, the role of polysaccharides in the treatment of RA has received widespread attention, and related studies have shown that polysaccharides can attenuate the immune response of RA by regulating the function and number of immune cells and balancing the immune system (59). However, although these studies have provided new ideas for the treatment of RA, there are also some problems that deserve in-depth discussion. Studies have shown that polysaccharides can enhance the activity of T-lymphocytes, B-lymphocytes and natural killer cells (NK cells), and promote the secretion of immune factors, while inhibiting excessive inflammatory responses (60, 61). For example, in a study that included 60 patients with RA and 40 patients with Huntington’s disease (HD), the microbial polysaccharide Galactoxylomannan (GalXM) was able to increase the activation of caspase-3 and ultimately increase the rate of apoptosis in T cells subset. GalXM was able to reduce STAT3 phosphorylation, IL-17 production and Th17 cell proliferation. It was also demonstrated that CD45 expression on target T cells was required to mediate the immunomodulatory effects of GAlXM (62). In terms of the studies themselves, most of the existing studies on the mechanisms of polysaccharides in RA therapy have focused on *in vitro* cellular experiments and animal models and small sample sizes. The limitation of sample size may lead to the bias of research results, and it is difficult to fully reflect the real efficacy and safety of GalXM in RA patients. In addition, there are differences between animal experiments and human physiological environments, and whether the mechanism of action of polysaccharides in animals is fully applicable to humans needs to be further verified (63). Guo et al. (13) prepared a novel self-assembled nanoparticle containing Celastrol (Cel) and matrix metalloproteinase (MMP)-sensitive chemo-acoustic kinetic therapies targeting macrophages at the site of inflammation in rheumatoid arthritis from Achyranthes polysaccharide. The final results show that DS-PVGLIG-Cel&Abps-thioketal-Cur@Cel nanomicelles (DPC&ATC@Cel) has a favorable *in vitro* and *in vivo* ability to treat RA with a good *in vivo* safety profile. Natural polysaccharides such as chitosan, alginate and hyaluronic acid are widely used in RA therapy because they are biocompatible and can be easily functionalized to enhance drug loading and targeting (64). However, in the human body, the targeting of nanoparticles, the *in vivo* metabolic process, and the safety of long-term use still need to be studied in depth (65). In a review, *Ganoderma lucidum* polysaccharides (GLP) inhibited the proliferation and migration of synovial fibroblasts (RASF) in RA, modulated pro- and anti-inflammatory cytokines, and reduced synovial inflammation. Secondly, GLP regulates the proliferation and differentiation of antigen-presenting cells such as dendritic cells, inhibits the phagocytosis of monocyte-derived macrophages and natural killer (NK) cells, and regulates the ratio of M1, M2 and related inflammatory cytokines. In addition, GLP produces activities that balance humoral and cellular immunity, such as regulating the production of immunoglobulins, the proliferative response of T and B lymphocytes and cytokine release, thus demonstrating immunomodulatory effects (43).

#### 4.2.2 Inflammation suppression

Polysaccharides have significant anti-inflammatory activity, inhibiting the infiltration of inflammatory cells and the release of inflammatory factors, and reducing joint swelling and pain (66, 67). In a study, different doses of *Angelica sinensis* polysaccharide (ASP) alleviated paw swelling in rat models of collagen-induced arthritis (CIA). Antibody levels in the ASP-treated group also showed varying degrees of reduction. Anti-CII IgG and anti-CII IgG2a levels were reduced in a dose-dependent manner, and ASP reduced the effects of CIA by attenuating TNF-α pro-inflammatory factors (68). In another study, administration of *Lycium barbarum* polysaccharide (LBP) significantly reduced serum IL-1α, IL-1β, IL-12, and IL-17 levels and restored and normalized IL-10 levels in a rat model of CIA. LBP reduced pro-inflammatory cytokine levels and restored anti-inflammatory cytokine levels (69). *Lonicerae Japonicae Caulis* polysaccharide (LJCP-2b) is a homogeneous heteropolysaccharide. Its structure mainly consisted of 1,3,6-β-D-Galp, 1,4-α-D-Glcp, 1,4,6-α-D-Glcp, 1,4-β-D-Galp, 1,2,4-α-L-Rhap and 1,4-α-D-GalpA. *In vitro* experiments demonstrated that LJCP-2b affected TNF-α-induced rheumatoid arthritis fibroblast-like synoviocyte (RA-FLS) functions, including attenuation of cell viability, increase in apoptosis, decrease in the number of migratory movements and adhesion capacity, and reduction in the levels of IL-6 and IL-1β. These results suggest that LJCP-2b has the activity to inhibit RA-FLS hyperproliferation and inflammatory response (70). Glycosaminoglycans (GAG) play a crucial role in the pathophysiology of RA (71). One of the main pathways by which these long-chain polysaccharides in the extracellular matrix and on the cell surface interact with growth factors, cytokines, and proteases that influence cell behavior is the TGF-β (transforming growth factor-β) signaling pathway, where GAGs enhance the anti-inflammatory effects of TGF-β, promote the differentiation of regulatory T-cells (Tregs), and inhibit pro-inflammatory cytokine production (72). In addition, Sun et al. extracted polysaccharides with anti-inflammatory activity from Large-leaf Yellow tea (LYT) and identified the presence of β-d-Xylp(1→, →2, 4)-β-d-Xylp(1→, →3)-β-d-Manp(1→, α-d-Glcp(1→ and →2, 4)-α-d-GalAp(1→ linkages. They found that LYT polysaccharides inhibited the migration and proliferation of MH7A cells and reduced NO production in a TNF-α-induced inflammation model. NO regulates the production of a variety of inflammatory factors, and its reduced production can upset the balance between pro- and anti-inflammatory factors, leading to difficulty in effectively controlling the inflammatory response and affecting immune homeostasis. The abundant presence of xylose accounts for 39% of the polysaccharide structure of LYT, and its unique linkage pattern (→2, 4)-β-d-Xylp(1→) appears to be a major contributor to its anti-inflammatory effects (73). Lin et al. introduced an alginate nanogel embedded in liposomes designed to enhance *in vivo* stability while retaining the inherent benefits of liposomes for RA. By incorporating an alginate network, the liposomes showed increased stiffness, reduced drug leakage, and improved cellular uptake by inflammatory macrophages. In addition, the encapsulated anti-inflammatory chlorogenic acid significantly inhibited ROS production and inflammatory response in arthritic rats, resulting in better therapeutic efficacy (74). Existing studies have relied on the CIA model, which mimics the acute inflammatory response but is fundamentally different from the chronic, progressive synovial lesions of human RA (75). The collagen-induced arthritis (CIA) model involves both cellular (particularly Th17-mediated) and humoral immune responses, and its inflammatory microenvironment involves more complex cell-cell interactions (76). Furthermore, whether the structural complexity of polysaccharides acts through multi-target regulation remains to be systematically evaluated.

#### 4.2.3 Ameliorating gut microbiota and its metabolites

Gut microbiota as one of the factors that can directly influence the body’s immune response, the role of gut microbiota in the field of RA has been gradually explored (77). Improving the gut microbiota and its metabolites can jointly have an impact on the development of diseases such as RA. Regulating the intestinal microbiota to restore its balanced state, as well as improving the composition and level of metabolites, can reduce the release of pro-inflammatory factors, inhibit the activation of inflammatory vesicles, etc., thereby alleviating the inflammatory response, relieving the symptoms of RA, and playing a protective role for the body (58). Some studies have shown that probiotics such as *Lactobacillus reuteri* can improve the symptoms of RA, which can be a promising novel target, with the ‘gut-joint’ axis as an important potential mechanism. The gut-joint axis refers to the stable, bi-directionally regulated interaction between the gut microbiota and the joints. Imbalances in the intestinal microbiota can affect the development of joint diseases such as RA. At the same time, joint diseases also change the structure and function of the gut microbiota (58, 78, 79). In the intestinal microbiota of RA patients, there is a significant increase in the abundance of pathogenic bacteria such as *Ruminococcus gnavus* group and a decrease in beneficial short-chain fatty acid (SCFAs) producing bacteria such as *Roseburia*, and these microbiota imbalances promote IL-6 by impairing antioxidant capacity, TNF-α and other pro-inflammatory factors release, exacerbating the development of RA (80). In addition, gut microbiota metabolites such as trimethylamine oxide (TMAO) in RA patients may promote synovial inflammation by activating NLRP3 inflammatory vesicles (81). For example, polysaccharides from Dianbaizhu (DBZP) treatment can affect the abundance of several specific bacteria in CIA mice, such as *Lactobacillus*, *Anaplasma* spp. *Alistipes*, *Enterorhabdus*, *Mucispirillum* and *Candidatus_Saccharimonas*, as well as a number of fecal or urinary Metabolites, such as 11β-hydroxytestosterone, pregnanediol 3-O-glucuronide, p-cresol sulfate and several amino acids and peptides, were also altered. The results suggest that DBZP has a protective effect on CIA in mice by modulating the gut microbiota (82). Linear discriminant analysis Effect Size (LEfSe) is one of the core tools for microbiome research by integrating non-parametric tests and effect size analysis to efficiently identify biomarkers of intergroup differences. In addition, researchers identified 12 bacterial strains enriched in *Acanthopanax senticosus* polysaccharide (ASPS)-treated mice and 2 strains enriched in CIA mice after initial immunization by LEfSe analysis. ASPS treatment significantly reversed the trend of increased abundance of *Bacteroides* and the ratio of *Bacteroidota/Bacillota* induced by the progression of arthritis. Similar results were observed at the class, order, and family levels finding *Acetatifactor*, *Ruminalococcus*, *Colidextribacter*, *Blautia*, and were significantly enriched at the genus level in ASPS-treated mice (83). The “gut-joint axis” theory of RA reveals that intestinal microbiota are involved in the pathogenesis of joint inflammation through bidirectional regulation, and polysaccharides have been shown to alleviate the symptoms of RA in mouse models by remodeling the structure of intestinal microbiota and metabolites (84). However, there may be fundamental differences in the regulatory targets of polysaccharides on different host microbiota. Different polysaccharides are preferentially degraded by specific groups of bacteria due to differences in glycosidic bond type and branching structure (85). *Segatella copri* is a bacterium widely found in the human gut (86). A variety of lactic acid bacteria convert primary bile acids to secondary bile acids via bile salt hydrolases, which activate farnesoid X receptor (FXR) and inhibit intestinal Th17 cells (87). *Segatella copri* lacks Bile Salt Hydrolase (BSH) activity and is unable to participate in bile acid metabolism. However, its overproliferation may indirectly weaken the anti-inflammatory pathway by decreasing the metabolic substrate for beneficial bacteria through consumption of bile salts (88). In addition, excessive inhibition of *Bacteroides* may affect dietary fiber metabolism, and enrichment of *Lactobacillus* may increase the risk of bloating in patients with irritable bowel syndrome (89). Therefore, future assessments regarding the intestinal tolerance of polysaccharide interventions need to be focused.

#### 4.2.4 Promotes cartilage and bone tissue repair

At the same time, polysaccharide can also intervene in the core signaling axis of RANKL/OPG (Key factor in the regulation of bone metabolism), remodel the microenvironment of immune-bone metabolism interaction to regulate the function of osteoclasts and osteoblasts, maintain the balance of bone metabolism, and promote the repair and regeneration of bone tissue (90). At the same time, polysaccharides can also regulate the functions of osteoclasts and osteoblasts, maintain the balance of bone metabolism, and promote the repair and regeneration of bone tissue (91). Ma et al. found that *Ephedra sinica* polysaccharide (ESP) treatment attenuated the significant infiltration of inflammatory cells, fibroblast proliferation and neovascularization, unevenness of articular cartilage surfaces, localized vascular shadowing, and damage to articular cartilage surfaces, and poorly defined borders of cartilage and subchondral tissues in ankle joints of mice in the CIA group (92). Similarly, the expression of osteogenesis-related genes (ALP and RUNX2) was significantly upregulated by *Sporidiobolus pararoseus* polysaccharides (SPP), which is essential for osteoblast differentiation and bone formation. On the other hand, the bone remodeling signaling pathway was stimulated by SPP, which significantly reduced the RANKL/OPG ratio and TRAF6 (Tumor necrosis factor receptor-related factor 6) expression, indicating reduced osteoclast activity and differentiation (93). The chemical structures of polysaccharides in the existing studies were roughly analyzed, such as the monosaccharide composition of ESP and the conformational relationship between bone protective activity were not clarified, and different extraction processes may lead to differences in the degree of sulfation modification (94), which directly affects its binding ability to RANKL. In addition, most of the trials in the efficacy assessment indexes were based on histopathologic scores. In contrast, human RA needs to be assessed by imaging hard endpoints for bone protection, such as X-ray Sharp score and MRI bone marrow edema score, which were completely missing in the existing studies.

#### 4.2.5 Polysaccharides alleviate RA through related signaling pathways

**Figure 5:**
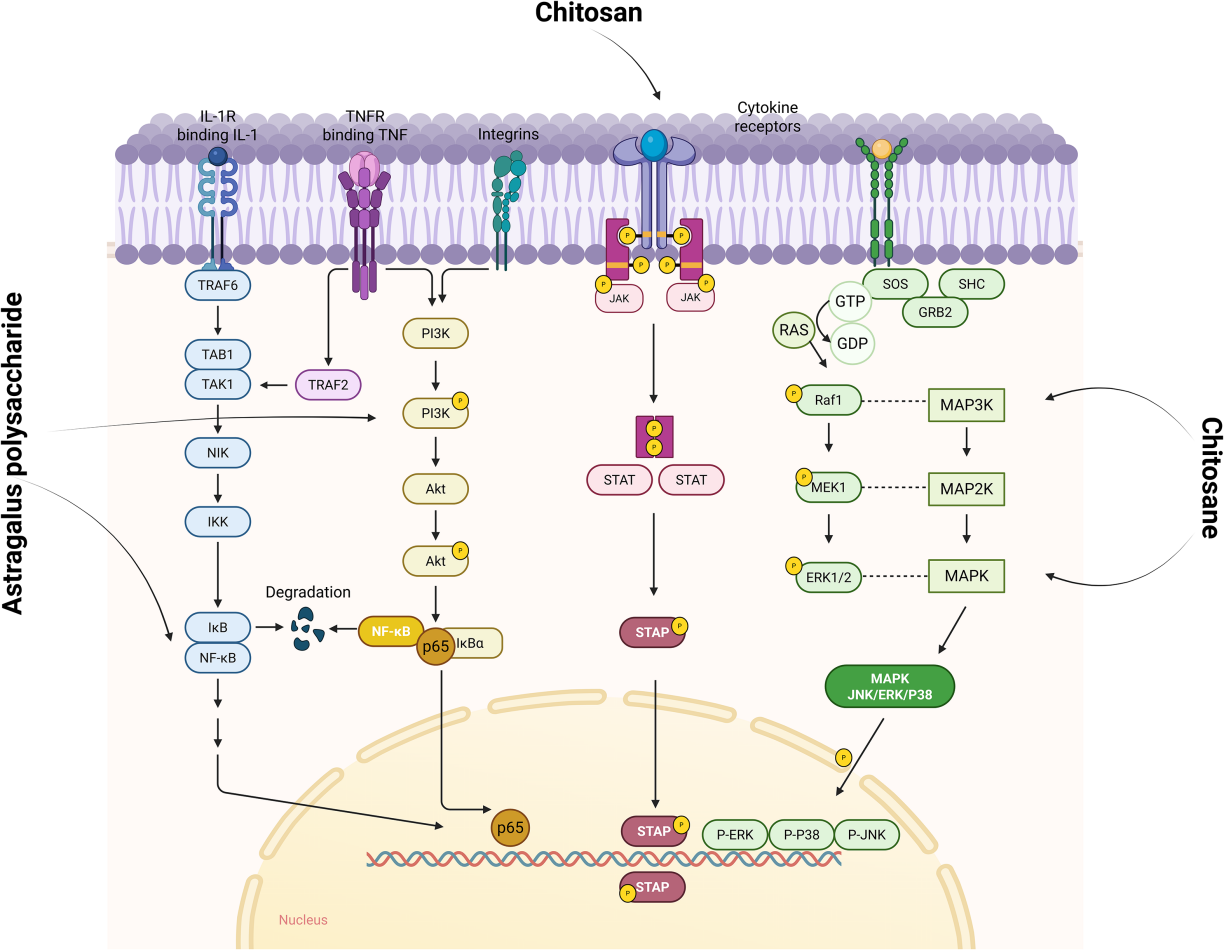
Polysaccharides affect signaling pathways such as NF-κB, PI3K/AKT, JAK/STAT, and MAPK to improve RA. IL-1R (interleukin-1 receptor) is a key upstream initiator of the NF-κB signaling pathway activating NF-κB through the TRAF6-TAK1-IKK cascade reaction, a central driver of the inflammatory response. While TNFR initiates the PI3K/AKT signaling pathway, it can also lead to phosphorylated degradation of IκBα through activation of the IKK complex, which in turn releases and activates NF-κB transcription factors. In the JAK/STAT signaling pathway, cytokine binding to the receptor activates JAK, which in turn catalyzes STAT phosphorylation, and the phosphorylated STAT forms a dimer that enters the nucleus to regulate gene expression. In the MAPK (including JNK, ERK, and P38) signaling pathway, upstream growth factors bind to the receptor to phosphorylate SHC, recruiting GRB2 and SOS. SOS prompts activation by exchanging Ras-bound GDP for GTP. Activated Ras activates Raf1 (a MAP3K), MAP3K activates MEK1 (MAP2K), and MEK1 phosphorylates ERK1/2 (MAPK). ERK enters the nucleus and regulates gene expression, while JNK and P38 function through a similar cascade. Image created with BioRender.com, with permission.

As shown in **Figure 5**, polysaccharides inhibit the activation of inflammatory signaling pathways such as NF-κB, PI3K/AKT, JAK/STAT and MAPK, and reduce the expression and release of inflammatory factors. By inhibiting the activation of inflammatory signaling pathways, polysaccharides reduce the inflammatory response of synovial tissues, thus alleviating the symptoms of RA. The occurrence of RA is closely related to the dysfunction of NF - κB, and the expression level of NF - κB in the lesion synovial tissue of RA patients is significantly increased. Highly activated NF - κB induces the production of various pro-inflammatory cytokines (such as TNF - α, IL-1 β and IL-6), and the pro-inflammatory cytokines regulate the activation of NF - κB through positive feedback, forming a vicious cycle to aggravate the progression of RA. Relevant studies have shown that the development of RA is closely related to NF-κB dysfunction, and the expression level of NF-κB is significantly elevated in the lesional synovial tissues of RA patients (95, 96). Highly activated NF-κB induces the production of various pro-inflammatory cytokines, such as tumour necrosis factor α (TNF-α), interleukin 1β (IL-1β), and IL-6, accelerating the progression of the disease. The upregulation of pro-inflammatory cytokines can in turn regulate the activation of NF-κB through positive feedback, forming a vicious circle and aggravating the progression of RA (97, 98). GAGs affect the Wnt/β-catenin pathway, which is essential for cartilage homeostasis and repair (99). GAGs are involved in the NF-κB signaling pathway, which reduces the activation of pro-inflammatory mediators, such as TNF-α and IL-1β, which both play a central role in RA. For example, administration of Astragalus polysaccharide (APS) to CIA rats reversed the expression levels of NF-κB-p65 and IκBα, thereby blocking the harmful feedback and loop (increase in pro-inflammatory cytokines→activation of NF-κB signaling→induced release of pro-inflammatory cytokines) (100). Similarly, Researchers investigated SD-a from *Saposhnikovia divaricata* (Trucz.) Schischk and investigated the molecular mechanism of the anti-rheumatoid effect of SD-a in a rat model of CIA. The results showed that SD-a could inhibit the significantly elevated expression of TLR4 and TRAF6 proteins in the CIA group, and significantly inhibited the phosphorylation of IκB-α and the nuclear translocation of NF-κB p65 (101). Chitosan reduces the expression of cytokines such as TNF-α, IL-1β and IL-6, which are central to the pathogenesis of RA, by inhibiting NF-κB activation (102).

The JAK/STAT pathway is one of the important signal transduction pathways of cytokines, regulating the growth, activation, differentiation, apoptosis and functions of cells. In the hematopoietic tissues of RA patients, JAK3, STAT and phosphorylated STAT are mainly highly expressed in activated T cells, B cells and FLS in the synovial lining layer. Chitosan also affects the JAK/STAT signaling pathway by modulating Janus kinase (JAK) activity, thereby reducing downstream transcription of inflammatory mediators (103). It has been reported that the phosphorylation levels of IκB and p65 in the NF-κB pathway in synovial tissues of CIA mice in the model group were increased to a very high level, whereas the phosphorylation levels in the Dendrobium huoshanense stem polysaccharide (cDHPS)-L group and the cDHPS-H group were reduced by 0-70%, respectively. 30-70%, respectively. Similarly, cDHPS also significantly reduced the elevated phosphorylation of JNK, p38, ERK1/2, PI3K, AKT, JAK1 and STAT3 in a dose-dependent manner in CIA mice. Apparently, cDHPS significantly inhibited the phosphorylation of IκB, p65, JNK, p38, ERK1/2, AKT, PI3K, JAK1 and STAT3 in CIA mice (104). The MAPK pathway, which contains components of JNK, p38, and ERK1/2, plays an important role in RA pathogenesis. In addition, chitosan has been shown to modulate the mitogen-activated protein kinase (MAPK) pathway, which influences cell proliferation, differentiation, and cytokine release in synovial tissue. This characteristic of chitosan oligosaccharides may have a potential adverse effect on bone homeostasis (105).

RANKL was abundantly expressed in the joint cavity of RA patients and combined with RANK on the surface of osteoclasts and osteoclast precursor cells, inducing the proliferation and differentiation of osteoclast precursor cells, increasing the activity of osteoclasts, and promoting bone resorption, as well as inhibiting the differentiation and function of osteoblasts. For example, the intervention of SPP significantly reduced rheumatoid factor, M1 macrophage activation and pro-inflammatory factors in CIA mice. Comprehensive metabolomics and gene expression analyses showed that SPP alleviated RA through arachidonic acid metabolism and OPG/RANKL/TRAF6 signaling pathway, played a key role in regulating metabolism and osteoblastic/osteoclastic gene expression in RA progression, and stimulated osteogenic remodeling (93). An *in vitro* and *in vivo* study of the therapeutic effects of ASP on RA showed that ASP inhibited TNF-α-induced phosphorylation of components of the JAK2/STAT3 and MAPK signaling pathways in CIA-FLS cells.ASP also inhibited inflammatory cytokine invasion and secretion via JAK2/STAT3 and MAPK signaling by FLS cells from CIA rats (106).

Existing studies have shown that polysaccharides often act through non-specific inhibition of multiple pathways, such as cDHPS, which simultaneously inhibits NF-κB-p65 nuclear translocation, JNK/p38/ERK phosphorylation and JAK1/STAT3 activation (104). However, the NF-κB pathway not only mediates inflammation, but also participates in apoptosis, and long-term inhibition may increase the risk of infection and tumor (107). The overall protein phosphorylation levels (p-NF-κB-p65, p-STAT3) in synovial tissues were mostly detected by Western blot in animal experiments, but there was a significant heterogeneity in pathway activation among different cell subpopulations in the synovial membranes of human RA (108).

## 5 Limitations

The review of the role and mechanisms of polysaccharides in the alleviation of RA does have some limitations. As a class of complex biomolecules, the specific mechanisms of action of polysaccharides have not been fully elucidated. Although some studies have shown that polysaccharides possess biological activities such as anti-inflammatory, antioxidant, and immunomodulatory activities, the specific mechanisms of these effects in RA still need further investigation. Therefore, this evaluation may not be able to elaborate in detail and accurately on the mechanism of action of polysaccharides in the treatment of RA *in vitro* and *in vivo* (in mice and humans). In addition, due to the differences in experimental conditions, animal models, polysaccharide sources and extraction methods, the experimental data derived from different studies may differ significantly. This makes it challenging for reviews to integrate and analyse these data and to draw consistent conclusions. Currently, polysaccharide studies have shown some efficacy in the animal and *in vitro* experimental stages, but the effects in clinical applications still need to be further verified.

## 6 Prospects and future directions

In the therapeutic exploration of RA, polysaccharides have demonstrated multifaceted potential and promise. In the field of targeted delivery systems, polysaccharides can deliver drugs directly, which not only ensures the sustained release of drugs, but also reduces the frequency of local drug delivery and toxic side effects, improves therapeutic safety, and provides a new idea for the development of highly efficient and low-toxicity drug delivery systems. In terms of synergistic combination therapy, dexamethasone in combination with hyssop polysaccharide has been shown to have better therapeutic effects on rheumatoid arthritis than either drug alone, reducing pathological symptoms, improving osteoporosis, and restoring athletic ability, which suggests that the combination of polysaccharides and other drugs is expected to play a synergistic role in providing a better solution for clinical treatment. In addition, in-depth investigation of the role of the gut microbiota-joint axis in the pathogenesis and therapeutic response of RA is also an important direction for the future. Studies have shown that intestinal microbiota dysbiosis is closely related to the development of RA, and polysaccharide vaccines can regulate the intestinal microbiota, maintain the homeostasis of intestinal microbiota, and prevent the autoimmune response caused by intestinal microbiota dysbiosis. Perhaps in the future, based on the modulation of the gut microbiota by polysaccharides, precise therapeutic strategies targeting the gut microbiota-joint axis could be developed. At present, no public information on ongoing clinical trials of polysaccharide in the treatment of RA has been retrieved, but as the research on polysaccharide in the treatment of RA continues to deepen, it is expected that more clinical trials will be conducted in the future to further validate the safety and efficacy of polysaccharide in the treatment of RA, and to promote the development of polysaccharide from basic research to clinical application, which will bring a new hope of treatment to the majority of patients with RA.

## 7 Conclusions

Polysaccharides, as a class of natural active ingredients, have shown therapeutic potential to alleviate RA by modulating immune responses, inhibiting inflammation, combating oxidative stress and promoting articular cartilage repair. However, the evidence for their use as stand-alone therapies is insufficient, and they are more often used as a complement to conventional therapies to synergize with mainstream drugs. In clinical application, it is necessary to strictly evaluate the patient’s condition, including metabolic status and intestinal microecology, to avoid blindly replacing standardized therapeutic regimens.

## References

[1] Gholijani N Azarpira N Abolmaali SS Tanideh N Ravanrooy MH Taki F. Piperine and piperine-loaded albumin nanoparticles ameliorate adjuvant-induced arthritis and reduce IL-17 in rats. Exp Mol Pathol. (2024) 140:104937. doi: 10.1016/j.yexmp.2024.104937 39353355

[2] Veale DJ Orr C Fearon U. Cellular and molecular perspectives in rheumatoid arthritis. Semin Immunopathol. (2017) 39:343–54. doi: 10.1007/s00281-017-0633-1 28508153

[3] Stainer A Tonutti A De Santis M Amati F Ceribelli A Bongiovanni G. Unmet needs and perspectives in rheumatoid arthritis-associated interstitial lung disease: A critical review. Front Med (Lausanne). (2023) 10:1129939. doi: 10.3389/fmed.2023.1129939 37007765 PMC10062456

[4] Liu S Liu J Cheng X Fang D Chen X Ding X. Application value of platelet-to-lymphocyte ratio as a novel indicator in rheumatoid arthritis: A review based on clinical evidence. J Inflammation Res. (2024) 17:7607–17. doi: 10.2147/JIR.S477262 PMC11512772 39464342

[5] Yi J Liu Y Xie H An H Li C Wang X. Hydrogels for the treatment of rheumatoid arthritis. Front bioengineering Biotechnol. (2022) 10:1014543. doi: 10.3389/fbioe.2022.1014543 PMC9597294 36312537

[6] Prasad P Verma S Surbhi Ganguly NK Chaturvedi V Mittal SA. Rheumatoid arthritis: advances in treatment strategies. Mol Cell Biochem. (2023) 478:69–88. doi: 10.1007/s11010-022-04492-3 35725992

[7] Jeong M Park JH. Nanomedicine for the treatment of rheumatoid arthritis. Mol pharmaceutics. (2021) 18:539–49. doi: 10.1021/acs.molpharmaceut.0c00295 32502346

[8] Wang Y Chen S Du K Liang C Wang S Owusu Boadi E. Traditional herbal medicine: Therapeutic potential in rheumatoid arthritis. J ethnopharmacology. (2021) 279:114368. doi: 10.1016/j.jep.2021.114368 34197960

[9] He YC Yao YM Xue QW Fang X Liang S. Anti-rheumatoid arthritis potential of diterpenoid fraction derived from Rhododendron molle fruits. Chin J Natural medicines. (2021) 19:181–7. doi: 10.1016/S1875-5364(21)60019-5 33781451

[10] Dai C Wang L You X Zhao Y Cao Z Wu J. Coffee-derived self-anti-inflammatory polymer as drug nanocarrier for enhanced rheumatoid arthritis treatment. Chin Chem Letters. (2025) 36:109869. doi: 10.1016/j.cclet.2024.109869

[11] Wijesinghe SN Lindsay MA Jones SW. Oligonucleotide therapies in the treatment of arthritis: A narrative review. Biomedicines. (2021) 9:902. doi: 10.3390/biomedicines9080902 34440106 PMC8389545

[12] Mo X Shen A Han Y Xu L Miao J Xu D. Polysaccharide nanoadjuvants engineered via phenotype-specific nanoprobe-assisted phenotypic screen reprogram macrophage cell functions for cancer and rheumatoid arthritis therapy. ACS nano. (2025) 19:12920–36. doi: 10.1021/acsnano.4c16671 PMC11984301 40152971

[13] Guo C Diao N Zhang D Cao M Wang W Geng H. Achyranthes polysaccharide based dual-responsive nano-delivery system for treatment of rheumatoid arthritis. Int J Biol macromolecules. (2023) 234:123677. doi: 10.1016/j.ijbiomac.2023.123677 36796562

[14] Noorbakhsh H Khorasgani MR. Date (Phoenix dactylifera L.) polysaccharides: a review on Chemical structure and nutritional properties. J Food Measurement Characterization. (2022) 16:3240–50. doi: 10.1007/s11694-022-01425-y

[15] El Asri S Ben Mrid R Zouaoui Z Roussi Z Ennoury A Nhiri M. Advances in structural modification of fucoidans, ulvans, and carrageenans to improve their biological functions for potential therapeutic application. Carbohydr Res. (2025) 549:109358. doi: 10.1016/j.carres.2024.109358 39718272

[16] Yin M Zhang Y Li H. Advances in research on immunoregulation of macrophages by plant polysaccharides. Front Immunol. (2019) 10:145. doi: 10.3389/fimmu.2019.00145 30804942 PMC6370632

[17] Qi M Zheng C Wu W Yu G Wang P. Exopolysaccharides from marine microbes: source, structure and application. Mar Drugs. (2022) 20:512. doi: 10.3390/md20080512 36005515 PMC9409974

[18] Liu S Hu J Zhong Y Hu X Yin J Xiong T. A review: Effects of microbial fermentation on the structure and bioactivity of polysaccharides in plant-based foods. Food Chem. (2024) 440:137453. doi: 10.1016/j.foodchem.2023.137453 38154284

[19] Paul S Parvez SS Goswami A Banik A. Exopolysaccharides from agriculturally important microorganisms: Conferring soil nutrient status and plant health. Int J Biol macromolecules. (2024) 262:129954. doi: 10.1016/j.ijbiomac.2024.129954 38336329

[20] Wang W Tan J Nima L Sang Y Cai X Xue H. Polysaccharides from fungi: A review on their extraction, purification, structural features, and biological activities. Food chemistry: X. (2022) 15:100414. doi: 10.1016/j.fochx.2022.100414 36211789 PMC9532758

[21] Shi Y Ma P. Pharmacological effects of Astragalus polysaccharides in treating neurodegenerative diseases. Front Pharmacol. (2024) 15:1449101. doi: 10.3389/fphar.2024.1449101 39156112 PMC11327089

[22] Yin Y Shi X Cai X Liu F Ni W Li B. Isolation techniques, structural characteristics, and pharmacological effects of phellinus polysaccharides: A review. Molecules (Basel Switzerland). (2024) 29:3047. doi: 10.3390/molecules29133047 38998999 PMC11243265

[23] Liu Y Shi Y Zou J Zhang X Zhai B Guo D. Extraction, purification, structural features, biological activities, modifications, and applications from Taraxacum mongolicum polysaccharides: A review. Int J Biol macromolecules. (2024) 259:129193. doi: 10.1016/j.ijbiomac.2023.129193 38191106

[24] McInnes IB Schett G. The pathogenesis of rheumatoid arthritis. New Engl J Med. (2011) 365:2205–19. doi: 10.1056/NEJMra1004965 22150039

[25] McInnes IB Schett G. Pathogenetic insights from the treatment of rheumatoid arthritis. Lancet (London England). (2017) 389:2328–37. doi: 10.1016/S0140-6736(17)31472-1 28612747

[26] Wang X Fan D Cao X Ye Q Wang Q Zhang M. The role of reactive oxygen species in the rheumatoid arthritis-associated synovial microenvironment. Antioxidants (Basel Switzerland). (2022) 11:1153. doi: 10.3390/antiox11061153 35740050 PMC9220354

[27] Lin Y Zhao YJ Zhang HL Hao WJ Zhu RD Wang Y. Regulatory role of KCa3.1 in immune cell function and its emerging association with rheumatoid arthritis. Front Immunol. (2022) 13:997621. doi: 10.3389/fimmu.2022.997621 36275686 PMC9580404

[28] Shen PC Huang SH Liu ZM Lu CC Chou SH Tien YC. Suramin ameliorates osteoarthritis by acting on the Nrf2/HO-1 and NF-κB signaling pathways in chondrocytes and promoting M2 polarization in macrophages. Int immunopharmacology. (2023) 120:110295. doi: 10.1016/j.intimp.2023.110295 37182454

[29] Jo HG Baek CY Hwang Y Baek E Park C Song HS. Investigating the anti-inflammatory, analgesic, and chondroprotective effects of gynostemma pentaphyllum (Thunb.) makino in osteoarthritis: an *in vitro* and *in vivo* study. Int J Mol Sci. (2024) 25:9594. doi: 10.3390/ijms25179594 39273553 PMC11395165

[30] Qi P Chen X Tian J Zhong K Qi Z Li M. The gut homeostasis-immune system axis: novel insights into rheumatoid arthritis pathogenesis and treatment. Front Immunol. (2024) 15:1482214. doi: 10.3389/fimmu.2024.1482214 39391302 PMC11464316

[31] Lu H Yao Y Yang J Zhang H Li L. Microbiome-miRNA interactions in the progress from undifferentiated arthritis to rheumatoid arthritis: evidence, hypotheses, and opportunities. Rheumatol Int. (2021) 41:1567–75. doi: 10.1007/s00296-021-04798-3 PMC8316166 33856544

[32] Zhang Z Wang G Zhang Z Liang X Wang G Xu M. Locally administered liposomal drug depot enhances rheumatoid arthritis treatment by inhibiting inflammation and promoting cartilage repair. J nanobiotechnology. (2025) 23:69. doi: 10.1186/s12951-025-03110-w 39891123 PMC11783794

[33] Blagov AV Grechko AV Nikiforov NG Zhuravlev AD Sadykhov NK Orekhov AN. Effects of metabolic disorders in immune cells and synoviocytes on the development of rheumatoid arthritis. Metabolites. (2022) 12:634. doi: 10.3390/metabo12070634 35888759 PMC9324614

[34] Scherer HU Häupl T Burmester GR. The etiology of rheumatoid arthritis. J autoimmunity. (2020) 110:102400. doi: 10.1016/j.jaut.2019.102400 31980337

[35] Tian X Wang Q Jiang N Zhao Y Huang C Liu Y. Chinese guidelines for the diagnosis and treatment of rheumatoid arthritis: 2024 update. Rheumatol Immunol Res. (2024) 5:189–208. doi: 10.1515/rir-2024-0028 39802551 PMC11720473

[36] England BR Smith BJ Baker NA Barton JL Oatis CA Guyatt G. 2022 American college of rheumatology guideline for exercise, rehabilitation, diet, and additional integrative interventions for rheumatoid arthritis. Arthritis Care Res. (2023) 75:1603–15. doi: 10.1002/acr.25117 37227116

[37] Mitrović J Hrkač S Tečer J Golob M Ljilja Posavec A Kolar Mitrović H. Pathogenesis of extraarticular manifestations in rheumatoid arthritis-A comprehensive review. Biomedicines. (2023) 11:1262. doi: 10.3390/biomedicines11051262. 37238933 PMC10216027

[38] Wang D Zhang J Lau J Wang S Taneja V Matteson EL. Mechanisms of lung disease development in rheumatoid arthritis. Nat Rev Rheumatol. (2019) 15:581–96. doi: 10.1038/s41584-019-0275-x 31455869

[39] Wu D Luo Y Li T Zhao X Lv T Fang G. Systemic complications of rheumatoid arthritis: Focus on pathogenesis and treatment. Front Immunol. (2022) 13:1051082. doi: 10.3389/fimmu.2022.1051082 36618407 PMC9817137

[40] Song X Wang YH Li MT Duan XW Li HB Zeng XF. Chinese registry of rheumatoid arthritis: IV. Correlation and consistency of rheumatoid arthritis disease activity indices in China. Chin Med J. (2021) 134:1465–70. doi: 10.1097/CM9.0000000000001517 PMC8443819 34134125

[41] Romero-Figueroa MDS Ramírez-Durán N Montiel-Jarquín AJ Horta-Baas G. Gut-joint axis: Gut dysbiosis can contribute to the onset of rheumatoid arthritis via multiple pathways. Front Cell infection Microbiol. (2023) 13:1092118. doi: 10.3389/fcimb.2023.1092118 PMC9911673 36779190

[42] He Y Huang Y Mai C Pan H Luo HB Liu L. The immunomodulatory role of PDEs inhibitors in immune cells: therapeutic implication in rheumatoid arthritis. Pharmacol Res. (2020) 161:105134. doi: 10.1016/j.phrs.2020.105134 32798648

[43] Meng M Yao J Zhang Y Sun H Liu M. Potential anti-rheumatoid arthritis activities and mechanisms of ganoderma lucidum polysaccharides. Molecules (Basel Switzerland). (2023) 28:2483. doi: 10.3390/molecules28062483 36985456 PMC10052150

[44] Suto T Tosevska A Dalwigk K Kugler M Dellinger M Stanic I. TNFR2 is critical for TNF-induced rheumatoid arthritis fibroblast-like synoviocyte inflammation. Rheumatol (Oxford England). (2022) 61:4535–46. doi: 10.1093/rheumatology/keac124 35258553

[45] Taghadosi M Adib M Jamshidi A Mahmoudi M Farhadi E. The p53 status in rheumatoid arthritis with focus on fibroblast-like synoviocytes. Immunologic Res. (2021) 69:225–38. doi: 10.1007/s12026-021-09202-7 33983569

[46] Kondo N Kuroda T Kobayashi D. Cytokine networks in the pathogenesis of rheumatoid arthritis. Int J Mol Sci. (2021) 22:10922. doi: 10.3390/ijms222010922 34681582 PMC8539723

[47] Lim JJ Jones CM Loh TJ Ting YT Zareie P Loh KL. The shared susceptibility epitope of HLA-DR4 binds citrullinated self-antigens and the TCR. Sci Immunol. (2021) 6:eabe0896. doi: 10.1126/sciimmunol.abe0896 33863750

[48] Erre GL Phan NDT Diaz N Congiargiu A Mundula N Mangoni AA. Microbial players in autoimmunity: multicentric analysis of the association between Mycoplasma hominis serostatus and rheumatoid arthritis. Microbiol spectrum. (2025) 13:e0147724. doi: 10.1128/spectrum.01477-24 PMC11878028 39902965

[49] Pathi A Wright M Smed MK Nelson JL Olsen J Hetland ML. The rheumatoid arthritis gene expression signature among women who improve or worsen during pregnancy: A pilot study. J Rheumatol. (2021) 48:985–91. doi: 10.3899/jrheum.201128 PMC8203750 33323535

[50] GianFrancesco MA Crowson CS. Where there’s smoke, there’s a joint: passive smoking and rheumatoid arthritis. Arthritis Rheumatol (Hoboken NJ). (2021) 73:2161–2. doi: 10.1002/art.41940 PMC8671157 34347946

[51] Smid DJ Klous L Ballak SB Catoire M De Hoogh IM Hoevenaars FPM. Exploring the role of nutritional strategies to influence physiological and cognitive mechanisms in cold weather operations in military personnel. Front Physiol. (2025) 16:1539615. doi: 10.3389/fphys.2025.1539615 40092148 PMC11907006

[52] Min YS Kim MG Ahn YS. Rheumatoid arthritis in silica-exposed workers. Int J Environ Res Public Health. (2021) 18:12776. doi: 10.3390/ijerph182312776 34886499 PMC8657481

[53] Zhong X Wang X Xu L Zhang J Yu W Ji L. Alterations in gut microbiota in Rheumatoid arthritis patients with interstitial lung Disease: A Comparative study. Hum Immunol. (2025) 86:111239. doi: 10.1016/j.humimm.2025.111239 39983663

[54] Shao T Hsu R Rafizadeh DL Wang L Bowlus CL Kumar N. The gut ecosystem and immune tolerance. J autoimmunity. (2023) 141:103114. doi: 10.1016/j.jaut.2023.103114 37748979

[55] Xu M Ren J Jiang Z Zhou S Wang E Li H. Structural characterization and immunostimulant activities of polysaccharides fractionated by gradient ethanol precipitation method from Panax ginseng C. A. Meyer. Front Pharmacol. (2024) 15:1388206. doi: 10.3389/fphar.2024.1388206 38720774 PMC11076722

[56] Li N Xu T Wu Z Zhao Y Ruan M Xu H. Arabinogalactan from Cynanchum atratum induces tolerogenic dendritic cells in gut to restrain autoimmune response and alleviate collagen-induced arthritis in mice. Phytomedicine. (2025) 136:156269. doi: 10.1016/j.phymed.2024.156269 39586124

[57] Li X Zhou Y Chen X Wang H Yang S Yang J. Semi-synthetic chondroitin sulfate CS-semi5 upregulates miR-122-5p, conferring a therapeutic effect on osteoarthritis via the p38/MMP13 pathway. Acta Pharm Sin B. (2024) 14:3528–42. doi: 10.1016/j.apsb.2024.05.016 PMC11365380 39220883

[58] Bodkhe R Balakrishnan B Taneja V. The role of microbiome in rheumatoid arthritis treatment. Ther Adv musculoskeletal Dis. (2019) 11:1759720x19844632. doi: 10.1177/1759720X19844632 PMC6685117 31431810

[59] Rong X Shen C Shu Q. Interplay between traditional Chinese medicine polysaccharides and gut microbiota: The elusive “polysaccharides-bond-bacteria-enzyme” equation. Phytotherapy research: PTR. (2024) 38:4695–715. doi: 10.1002/ptr.8284 39120443

[60] Shibuya A Shibuya K. DNAM-1 versus TIGIT: competitive roles in tumor immunity and inflammatory responses. Int Immunol. (2021) 33:687–92. doi: 10.1093/intimm/dxab085 34694361

[61] Cai G Wu C Zhu T Peng S Xu S Hu Y. Structure of a Pueraria root polysaccharide and its immunoregulatory activity on T and B lymphocytes, macrophages, and immunosuppressive mice. Int J Biol macromolecules. (2023) 230:123386. doi: 10.1016/j.ijbiomac.2023.123386 36702224

[62] Alunno A Pericolini E Gabrielli E Bistoni O Caterbi S Bartoloni E. THU0129 Selective elimination of pathogenic TH17 cells from peripheral blood of rheumatoid arthritis patients by a purified fungal polysaccharide. Ann Rheumatic Diseases. (2012) 71:198. doi: 10.1136/annrheumdis-2012-eular.2094

[63] Van Norman GA. Limitations of animal studies for predicting toxicity in clinical trials: is it time to rethink our current approach? JACC Basic Transl Sci. (2019) 4:845–54. doi: 10.1016/j.jacbts.2019.10.008 PMC6978558 31998852

[64] Serrano-Sevilla I Artiga Á Mitchell SG De Matteis L de la Fuente JM. Natural polysaccharides for siRNA delivery: nanocarriers based on chitosan, hyaluronic acid, and their derivatives. Molecules (Basel Switzerland). (2019) 24:2570. doi: 10.3390/molecules24142570 31311176 PMC6680562

[65] Fernandes Q Billa N. Amygdalin in antineoplastic medicine and the relevance of nanotechnology. BioMed Pharmacother. (2025) 182:117772. doi: 10.1016/j.biopha.2024.117772 39700870

[66] Alenazi F Moursi S Mahmoud MR Shahid SMA Khatoon F Shahid Khan M. Withaferin A alleviates inflammation in animal models of arthritis by inhibiting the NF-κB pathway and cytokine release. Chemico-biological interactions. (2024) 398:111114. doi: 10.1016/j.cbi.2024.111114 38897341

[67] Huang D Jiang S Du Z Chen Y Xue D Wang X. Analgesic and anti-arthritic activities of polysaccharides in chaenomeles speciosa. Front Pharmacol. (2022) 13:744915. doi: 10.3389/fphar.2022.744915 35401173 PMC8989029

[68] Hu Q Wu C Yu J Luo J Peng X. Angelica sinensis polysaccharide improves rheumatoid arthritis by modifying the expression of intestinal Cldn5, Slit3 and Rgs18 through gut microbiota. Int J Biol macromolecules. (2022) 209:153–61. doi: 10.1016/j.ijbiomac.2022.03.090 35318077

[69] Lai W Wang C Lai R Peng X Luo J. Lycium barbarum polysaccharide modulates gut microbiota to alleviate rheumatoid arthritis in a rat model. NPJ Sci Food. (2022) 6:34. doi: 10.1038/s41538-022-00149-z 35864275 PMC9304368

[70] Bi Z Zhao Y Hu J Ding J Yang P Liu Y. A novel polysaccharide from Lonicerae Japonicae Caulis: Characterization and effects on the function of fibroblast-like synoviocytes. Carbohydr polymers. (2022) 292:119674. doi: 10.1016/j.carbpol.2022.119674 35725209

[71] Xu XL Shu GF Wang XJ Qi J Jin FY Shen QY. Sialic acid-modified chitosan oligosaccharide-based biphasic calcium phosphate promote synergetic bone formation in rheumatoid arthritis therapy. J Control Release. (2020) 323:578–90. doi: 10.1016/j.jconrel.2020.04.047 32376462

[72] Iacob S Cs-Szabo G. Biglycan regulates the expression of EGF receptors through EGF signaling pathways in human articular chondrocytes. Connect Tissue Res. (2010) 51:347–58. doi: 10.3109/03008200903427695 20367117

[73] Sun Q Du J Wang Z Li X Fu R Liu H. Structural characteristics and biological activity of a water-soluble polysaccharide HDCP-2 from Camellia sinensis. Int J Biol macromolecules. (2024) 277:134437. doi: 10.1016/j.ijbiomac.2024.134437 39116965

[74] Lin X Li Y Zhang B Li J Ren J Tang Y. Alginate nanogel-embedded liposomal drug carriers facilitate drug delivery efficiency in arthritis treatment. Int J Biol macromolecules. (2024) 273:133065. doi: 10.1016/j.ijbiomac.2024.133065 38866273

[75] de Molon RS Thurlings RM Walgreen B Helsen MM van der Kraan PM Cirelli JA. Systemic resolvin E1 (RvE1) treatment does not ameliorate the severity of collagen-induced arthritis (CIA) in mice: A randomized, prospective, and controlled proof of concept study. Mediators Inflamm. (2019) 2019:5689465. doi: 10.1155/2019/5689465 31780864 PMC6875002

[76] Yao F Zhao Y Yu Q Hu W Lin Y Chen Y. Extracellular CIRP induces abnormal activation of fibroblast-like synoviocytes from patients with RA via the TLR4-mediated HDAC3 pathways. Int immunopharmacology. (2024) 128:111525. doi: 10.1016/j.intimp.2024.111525 38218010

[77] Wang H Cai Y Wu W Zhang M Dai Y Wang Q. Exploring the role of gut microbiome in autoimmune diseases: A comprehensive review. Autoimmun Rev. (2024) 23:103654. doi: 10.1016/j.autrev.2024.103654 39384149

[78] Fujimoto K Uematsu S. Vaccine therapy for dysbiosis-related diseases. World J gastroenterology. (2020) 26:2758–67. doi: 10.3748/wjg.v26.i21.2758 PMC7284185 32550752

[79] Asoudeh F Djafarian K Akhalghi M Mahmoudi M Jamshidi AR Farhadi E. The effect of probiotic cheese consumption on inflammatory and anti-inflammatory markers, disease severity, and symptoms in patients with rheumatoid arthritis: study protocol for a randomized, double-blind, placebo-controlled trial. Trials. (2022) 23:180. doi: 10.1186/s13063-022-06113-2 35209942 PMC8876752

[80] Liu Z Wu Y Luo Y Wei S Lu C Zhou Y. Self-balance of intestinal flora in spouses of patients with rheumatoid arthritis. Front Med (Lausanne). (2020) 7:538. doi: 10.3389/fmed.2020.00538 33681234 PMC7931358

[81] Li X Geng J Zhao J Ni Q Zhao C Zheng Y. Trimethylamine N-oxide exacerbates cardiac fibrosis via activating the NLRP3 inflammasome. Front Physiol. (2019) 10:866. doi: 10.3389/fphys.2019.00866 31354519 PMC6634262

[82] Dong Y Wang Y Zhang F Ma J Li M Liu W. Polysaccharides from Gaultheria leucocarpa var. yunnanensis (DBZP) alleviates rheumatoid arthritis through ameliorating gut microbiota. Int J Biol macromolecules. (2024) 281:136250. doi: 10.1016/j.ijbiomac.2024.136250 39482128

[83] Liu A Zhang M Wu Y Zhang C Zhang Q Su X. ASPS exhibits anti-rheumatic effects by reprogramming gut microbiota and increasing serum γ-glutamylcysteine level. Advanced Sci (Weinheim Baden-Wurttemberg Germany). (2023) 10:e2205645. doi: 10.1002/advs.202205645 PMC9875676 36417588

[84] Li X Qin Y Yue F Lü X. Comprehensive analysis of fecal microbiome and metabolomics uncovered dl-norvaline-ameliorated obesity-associated disorders in high-fat diet-fed obese mice by targeting the gut microbiota. J Agric Food Chem. (2025) 73:2381–92. doi: 10.1021/acs.jafc.4c06638 39808000

[85] Xue H Liang B Wang Y Gao H Fang S Xie K. The regulatory effect of polysaccharides on the gut microbiota and their effect on human health: A review. Int J Biol macromolecules. (2024) 270:132170. doi: 10.1016/j.ijbiomac.2024.132170 38734333

[86] Blanco-Míguez A Gálvez EJC Pasolli E De Filippis F Amend L Huang KD. Extension of the Segatella copri complex to 13 species with distinct large extrachromosomal elements and associations with host conditions. Cell Host Microbe. (2023) 31:1804–19.e9. doi: 10.1016/j.chom.2023.09.013 37883976 PMC10635906

[87] Olotu T Ferrell JM. Lactobacillus sp. for the attenuation of metabolic dysfunction-associated steatotic liver disease in mice. Microorganisms. (2024) 12:2488. doi: 10.3390/microorganisms12122488 39770690 PMC11728176

[88] Wang L Hu R Ma S Yang X Gong J Xiang H. Dihydroquercetin attenuated Prevotella copri-caused intestinal injury by modulating gut microbiota and bile acids in weaned piglets. Anim Nutr. (2025) 20:303–10. doi: 10.1016/j.aninu.2024.10.002 PMC11849659 39995524

[89] Kwon H Nam EH Kim H Jo H Bang WY Lee M. Effect of Lacticaseibacillus rhamnosus IDCC 3201 on irritable bowel syndrome with constipation: a randomized, double-blind, and placebo-controlled trial. Sci Rep. (2024) 14:22384. doi: 10.1038/s41598-024-72887-x 39333245 PMC11437119

[90] Bourdon B Cassé F Gruchy N Cambier P Leclercq S Oddoux S. Marine collagen hydrolysates promote collagen synthesis, viability and proliferation while downregulating the synthesis of pro-catabolic markers in human articular chondrocytes. Int J Mol Sci. (2021) 22:3693. doi: 10.3390/ijms22073693 33916312 PMC8036580

[91] Kim S-H Park KH Lee J Lee SH Baek J-H. The effect of Schizophyllan on the differentiation of osteoclasts and osteoblasts. Biochem Biophys Res Commun. (2024) 710:149860. doi: 10.1016/j.bbrc.2024.149860 38604070

[92] Ma Y Wei X Peng J Wei F Wen Y Liu M. Ephedra sinica polysaccharide regulate the anti-inflammatory immunity of intestinal microecology and bacterial metabolites in rheumatoid arthritis. Front Pharmacol. (2024) 15:1414675. doi: 10.3389/fphar.2024.1414675 38846095 PMC11153800

[93] Zhu H Shen F Liao T Qian H Liu Y. Sporidiobolus pararoseus polysaccharides relieve rheumatoid arthritis by regulating arachidonic acid metabolism and bone remodeling signaling pathway. Int J Biol macromolecules. (2024) 281:136272. doi: 10.1016/j.ijbiomac.2024.136272 39366615

[94] Guo H Li HY Liu L Wu CY Liu H Zhao L. Effects of sulfated modification on the physicochemical properties and biological activities of β-glucans from Qingke (Tibetan hulless barley). Int J Biol macromolecules. (2019) 141:41–50. doi: 10.1016/j.ijbiomac.2019.08.245 31476391

[95] Shi L Zhao Y Feng C Miao F Dong L Wang T. Therapeutic effects of shaogan fuzi decoction in rheumatoid arthritis: Network pharmacology and experimental validation. Front Pharmacol. (2022) 13:967164. doi: 10.3389/fphar.2022.967164 36059943 PMC9428562

[96] Shen Y Teng L Qu Y Liu J Zhu X Chen S. Anti-proliferation and anti-inflammation effects of corilagin in rheumatoid arthritis by downregulating NF-κB and MAPK signaling pathways. J ethnopharmacology. (2022) 284:114791. doi: 10.1016/j.jep.2021.114791 34737112

[97] Liao H Zheng J Lu J Shen HL. NF-κB signaling pathway in rheumatoid arthritis: mechanisms and therapeutic potential. Mol Neurobiol. (2025) 62:6998–7021. doi: 10.1007/s12035-024-04634-2 39560902

[98] Ilchovska DD Barrow DM. An Overview of the NF-kB mechanism of pathophysiology in rheumatoid arthritis, investigation of the NF-kB ligand RANKL and related nutritional interventions. Autoimmun Rev. (2021) 20:102741. doi: 10.1016/j.autrev.2020.102741 33340772

[99] Praxenthaler H Krämer E Weisser M Hecht N Fischer J Grossner T. Extracellular matrix content and WNT/β-catenin levels of cartilage determine the chondrocyte response to compressive load. Biochim Biophys Acta Mol Basis Dis. (2018) 1864:851–9. doi: 10.1016/j.bbadis.2017.12.024 29277327

[100] Cao L Yu M Wang C Bao Y Zhang M He P. Cellulase-assisted extraction, characterization, and bioactivity against rheumatoid arthritis of astragalus polysaccharides. Int J Polymer Science. (2019) 2019:8514247. doi: 10.1155/2019/8514247

[101] Sun X Zhang T Liu S Zhao Y Sun X. The prepared and characterized polysaccharide polymer in Saposhnikovia divaricata(Trucz.) Schischk effectively controls the course of rheumatoid arthritis via TLR4/TRAF6–NF-κB/IκB-α signaling pathway. Biomedicine Pharmacotherapy. (2023) 160:114416. doi: 10.1016/j.biopha.2023.114416

[102] Xu L Qin J Ma X Wang Q Wu W Huang H. Chitosan-based self-healing thermosensitive hydrogel loaded with siHMGB1 for treatment of rheumatoid arthritis via macrophage repolarization. Int J Biol macromolecules. (2024) 282:137102. doi: 10.1016/j.ijbiomac.2024.137102 39486712

[103] Kong Y Zhang Y Zhao X Wang G Liu Q. Carboxymethyl-chitosan attenuates inducible nitric oxide synthase and promotes interleukin-10 production in rat chondrocytes. Exp Ther Med. (2017) 14:5641–6. doi: 10.3892/etm.2017.5258 PMC5740727 29285104

[104] Shang ZZ Qin DY Li QM Zha XQ Pan LH Peng DY. Dendrobium huoshanense stem polysaccharide ameliorates rheumatoid arthritis in mice via inhibition of inflammatory signaling pathways. Carbohydr polymers. (2021) 258:117657. doi: 10.1016/j.carbpol.2021.117657 33593544

[105] Bai BL Xie ZJ Weng SJ Wu ZY Li H Tao ZS. Chitosan oligosaccharide promotes osteoclast formation by stimulating the activation of MAPK and AKT signaling pathways. J Biomater Sci Polym Ed. (2018) 29:1207–18. doi: 10.1080/09205063.2018.1448336 29502489

[106] Xue Y Zhou S Yang Z Hao P Wang L Cui W. Angelica sinensis polysaccharide inhibits inflammation of collagen-induced arthritis rat fibroblast-like synoviocytes by inhibiting JAK2/STAT3 and MAPK signaling. Arabian J Chem. (2023) 16:105320. doi: 10.1016/j.arabjc.2023.105320

[107] Lalle G Twardowski J Grinberg-Bleyer Y. NF-κB in cancer immunity: friend or foe? Cells. (2021) 10:355. doi: 10.3390/cells10020355 33572260 PMC7914614

[108] MaChado CRL Dias FF Resende GG Oliveira PG Xavier RM Andrade MVM. Morphofunctional analysis of fibroblast-like synoviocytes in human rheumatoid arthritis and mouse collagen-induced arthritis. Adv Rheumatol. (2023) 63:1. doi: 10.1186/s42358-022-00281-0 36597166

[109] Jiang Q Yang G Liu Q Wang S Cui D. Function and role of regulatory T cells in rheumatoid arthritis. Front Immunol. (2021) 12:626193. doi: 10.3389/fimmu.2021.626193 33868244 PMC8047316

[110] Wu F Gao J Kang J Wang X Niu Q Liu J. B cells in rheumatoid arthritis:Pathogenic mechanisms and treatment prospects. Front Immunol. (2021) 12:750753. doi: 10.3389/fimmu.2021.750753 34650569 PMC8505880

[111] Baasch S Ruzsics Z Henneke P. Cytomegaloviruses and macrophages-friends and foes from early on? Front Immunol. (2020) 11:793. doi: 10.3389/fimmu.2020.00793 32477336 PMC7235172

[112] Edilova MI Akram A Abdul-Sater AA. Innate immunity drives pathogenesis of rheumatoid arthritis. Biomed J. (2021) 44:172–82. doi: 10.1016/j.bj.2020.06.010 PMC8178572 32798211

[113] Karmakar U Vermeren S. Crosstalk between B cells and neutrophils in rheumatoid arthritis. Immunology. (2021) 164:689–700. doi: 10.1111/imm.13412 34478165 PMC8561113

[114] Kim KW Kim BM Won JY Min HK Lee KA Lee SH. Regulation of osteoclastogenesis by mast cell in rheumatoid arthritis. Arthritis Res Ther. (2021) 23:124. doi: 10.1186/s13075-021-02491-1 33882986 PMC8059019

[115] Yan Y Lu A Dou Y Zhang Z Wang XY Zhai L. Nanomedicines reprogram synovial macrophages by scavenging nitric oxide and silencing CA9 in progressive osteoarthritis. Advanced Sci (Weinheim Baden-Wurttemberg Germany). (2023) 10:e2207490. doi: 10.1002/advs.202207490 PMC10104675 36748885

[116] Németh T Nagy G Pap T. Synovial fibroblasts as potential drug targets in rheumatoid arthritis, where do we stand and where shall we go? Ann Rheum Dis. (2022) 81:1055–64. doi: 10.1136/annrheumdis-2021-222021 PMC9279838 35715191

[117] Vial G Lambert C Pereira B Couderc M Malochet-Guinamand S Mathieu S. The effect of TNF and non-TNF-targeted biologics on body composition in rheumatoid arthritis. J Clin Med. (2021) 29:487. doi: 10.3390/jcm10030487 PMC7866419 33573047

[118] Pandolfi F Franza L Carusi V Altamura S Andriollo G Nucera E. Interleukin-6 in rheumatoid arthritis. Int J Mol Sci. (2020) 21:5238. doi: 10.3390/ijms21155238 32718086 PMC7432115

[119] Zhao R Zhang YW Yao JY Qiao J Song S Zhang SX. Genetic association between interleukin-17 and susceptibility to rheumatoid arthritis. BMC Med Genomics. (2023) 16:277. doi: 10.1186/s12920-023-01713-6 37926850 PMC10626638

[120] Han L Tu S Shen P Yan J Huang Y Ba X. A comprehensive transcriptomic analysis of alternate interferon signaling pathways in peripheral blood mononuclear cells in rheumatoid arthritis. Aging. (2021) 13:20511–33. doi: 10.18632/aging.203432 PMC8436925 34432649

[121] Hatipoğlu M Daltaban Ö Uğur S Üstün K Kaçar C Tuncer T. B cell depletion in patients with rheumatoid arthritis is associated with reduced IL-1β in GCF. Clin Oral investigations. (2022) 26:4307–13. doi: 10.1007/s00784-022-04378-0 35578115

[122] Min HK Kim S Lee JY Kim KW Lee SH Kim HR. IL-18 binding protein suppresses IL-17-induced osteoclastogenesis and rectifies type 17 helper T cell/regulatory T cell imbalance in rheumatoid arthritis. J Trans Med. (2021) 19:392. doi: 10.1186/s12967-021-03071-2 PMC8444577 34530864

[123] de la Aleja AG Herrero C Torres-Torresano M Schiaffino MT Del Castillo A Alonso B. Inhibition of LXR controls the polarization of human inflammatory macrophages through upregulation of MAFB. Cell Mol Life sciences: CMLS. (2023) 80:96. doi: 10.1007/s00018-023-04745-4 PMC10020776 36930354

[124] Wang Y Han J Yue Y Wu Y Zhang W Xia W. Purification, structure identification and immune activity of a neutral polysaccharide from Cynanchum Auriculatum. Int J Biol macromolecules. (2023) 237:124142. doi: 10.1016/j.ijbiomac.2023.124142 36972816

[125] Wei H Shi Y Yuan Z Huang Z Cai F Zhu J. Isolation, identification, and anti-inflammatory activity of polysaccharides of typha angustifolia. Biomacromolecules. (2021) 22:2451–9. doi: 10.1021/acs.biomac.1c00235 34024108

[126] ShanChen Khan BM Cheong KL Liu Y. Pumpkin polysaccharides: Purification, characterization and hypoglycemic potential. Int J Biol macromolecules. (2019) 139:842–9. doi: 10.1016/j.ijbiomac.2019.08.053 31400422

[127] Huang Z Zong M-H Lou W-Y. Preparation, structural elucidation and immunomodulatory activity of a polysaccharide from Millettia Speciosa Champ. Ind Crops Products. (2022) 182:114889. doi: 10.1016/j.indcrop.2022.114889

[128] Zhou R Cui M Wang Y Zhang M Li F Liu K. Isolation, structure identification and anti-inflammatory activity of a polysaccharide from Phragmites rhizoma. Int J Biol macromolecules. (2020) 161:810–7. doi: 10.1016/j.ijbiomac.2020.06.124 32553949

[129] Wu J Chen X Qiao K Su Y Liu Z. Purification, structural elucidation, and *in vitro* antitumor effects of novel polysaccharides from Bangia fuscopurpurea. Food Sci Hum Wellness. (2021) 10:63–71. doi: 10.1016/j.fshw.2020.05.003

[130] Jiang Y Shang Z Lv X Du M Ma L Hou G. Structure elucidation and antitumor activity of a water soluble polysaccharide from Hemicentrotus pulcherrimus. Carbohydr polymers. (2022) 292:119718. doi: 10.1016/j.carbpol.2022.119718 35725190

[131] Li S Zhong W Pan Y Lin L Cai Y Mao H. Structural characterization and anticoagulant analysis of the novel branched fucosylated glycosaminoglycan from sea cucumber Holothuria nobilis. Carbohydr polymers. (2021) 269:118290. doi: 10.1016/j.carbpol.2021.118290 34294316

[132] Ustyuzhanina NE Bilan MI Dmitrenok AS Tsvetkova EA Nifantiev NE Usov AI. Oversulfated dermatan sulfate and heparinoid in the starfish Lysastrosoma anthosticta: Structures and anticoagulant activity. Carbohydr polymers. (2021) 261:117867. doi: 10.1016/j.carbpol.2021.117867 33766355

[133] Xiang X-W Wang R Chen H Chen Y-F Shen G-X Liu S-L. Structural characterization of a novel marine polysaccharide from mussel and its antioxidant activity in RAW264.7 cells induced by H2O2. Food Bioscience. (2022) 47:101659. doi: 10.1016/j.fbio.2022.101659

[134] Du Z Jia X Chen J Zhou S Chen J Liu X. Isolation and characterization of a heparin-like compound with potent anticoagulant and fibrinolytic activity from the clam coelomactra antiquata. Mar Drugs. (2019) 18:6. doi: 10.3390/md18010006 31861572 PMC7024239

[135] Sheng Z Wen L Yang B. Structure identification of a polysaccharide in mushroom Lingzhi spore and its immunomodulatory activity. Carbohydr polymers. (2022) 278:118939. doi: 10.1016/j.carbpol.2021.118939 34973757

[136] Liang Z Zheng K Zhao Q Shao W Li C Wang J. Structural identification and coagulation effect of flammulina velutipes polysaccharides. Appl Sci [Internet]. (2021) 11:1736. doi: 10.3390/app11041736

[137] Yang X Lin P Wang J Liu N Yin F Shen N. Purification, characterization and anti-atherosclerotic effects of the polysaccharides from the fruiting body of Cordyceps militaris. Int J Biol macromolecules. (2021) 181:890–904. doi: 10.1016/j.ijbiomac.2021.04.083 33878353

[138] Dong M Hou Y Ding X. Structure identification, antitumor activity and mechanisms of a novel polysaccharide from Ramaria flaccida (Fr.) Quél. Oncol Lett. (2020) 20:2169–82. doi: 10.3892/ol.2020.11761 PMC7400858 32782534

[139] Su S Ding X Hou Y Liu B Du Z Liu J. Structure elucidation, immunomodulatory activity, antitumor activity and its molecular mechanism of a novel polysaccharide from Boletus reticulatus Schaeff. Food Sci Hum Wellness. (2023) 12:647–61. doi: 10.1016/j.fshw.2022.07.067

[140] Wang Q Shu Z Xing N Xu B Wang C Sun G. A pure polysaccharide from Ephedra sinica treating on arthritis and inhibiting cytokines expression. Int J Biol macromolecules. (2016) 86:177–88. doi: 10.1016/j.ijbiomac.2016.01.010 26835987

[141] Luo J Yang Q Jiang W Liu Y Hu Q Peng X. The interaction between Angelica sinensis polysaccharide ASP-2pb and specific gut bacteria alleviates rheumatoid arthritis in rats. Int J Biol macromolecules. (2025) 301:140473. doi: 10.1016/j.ijbiomac.2025.140473 39889994

[142] Guo Y Ye Q Yang S Wu J Ye B Wu Y. Therapeutic effects of polysaccharides from Anoectochilus roxburghii on type II collagen-induced arthritis in rats. Int J Biol macromolecules. (2019) 122:882–92. doi: 10.1016/j.ijbiomac.2018.11.015 30408452

[143] Zhao F Ma T Zhang X Zhao Q Zhu K Cao J. Holothuria leucospilota polysaccharides improve immunity and the gut microbiota in cyclophosphamide-treated immunosuppressed mice. Mol Nutr Food Res. (2023) 67:e2200317. doi: 10.1002/mnfr.202200317 36401832

[144] Liao T Shen F Zhu H Mu W Qian H Liu Y. Extracellular polysaccharides from Sporidiobolus pararoseus alleviates rheumatoid through ameliorating gut barrier function and gut microbiota. Int J Biol macromolecules. (2024) 260:129436. doi: 10.1016/j.ijbiomac.2024.129436 38228197

[145] Yang D Cheng X Fan M Xie D Liu Z Zheng F. Regulation of polysaccharide in Wu-tou decoction on intestinal microflora and pharmacokinetics of small molecular compounds in AIA rats. Chin Med. (2024) 19:9. doi: 10.1186/s13020-024-00878-1 38218825 PMC10787407

[146] Yang R Yan L Xu T Zhang K Lu X Xie C. Injectable bioadhesive hydrogel as a local nanomedicine depot for targeted regulation of inflammation and ferroptosis in rheumatoid arthritis. Biomaterials. (2024) 311:122706. doi: 10.1016/j.biomaterials.2024.122706 39032219

[147] Hannan A Akhtar B Sharif A Anjum F Pasha I Khan A. Quercetin-loaded chitosan nanoparticles ameliorate adjuvant-induced arthritis in rats by regulating anti-oxidant enzymes and downregulating pro- and inflammatory cytokines. Inflammopharmacology. (2023) 31:287–300. doi: 10.1007/s10787-022-01118-4 36542211

[148] Wang X Liu D Li D Yan J Yang J Zhong X. Combined treatment with glucosamine and chondroitin sulfate improves rheumatoid arthritis in rats by regulating the gut microbiota. Nutr Metab (Lond). (2023) 20:22. doi: 10.1186/s12986-023-00735-2 37016458 PMC10071728

